# A Role for the PERIOD:PERIOD Homodimer in the *Drosophila* Circadian Clock

**DOI:** 10.1371/journal.pbio.1000003

**Published:** 2009-04-28

**Authors:** Johannes Landskron, Ko Fan Chen, Eva Wolf, Ralf Stanewsky

**Affiliations:** 1 Institut für Zoologie, Universität Regensburg, Regensburg, Germany; 2 School of Biological and Chemical Sciences, Queen Mary, University of London, London, United Kingdom; 3 Max-Planck-Institute of Molecular Physiology, Dortmund, Germany; Rockefeller University, United States of America

## Abstract

Circadian clocks in eukaryotes rely on transcriptional feedback loops, in which clock genes repress their own transcription resulting in molecular oscillations with a period of ∼24 h. In *Drosophila*, the clock proteins Period (PER) and Timeless (TIM) operate in such a feedback loop, whereby they first accumulate in the cytoplasm of clock cells as a heterodimer. Nuclear translocation of the complex or the individual PER and TIM proteins is followed by repression of per and tim transcription, whereby PER seems to act as the prime repressor. We found that in addition to PER:TIM complexes, functional PER:PER homodimers exist in flies. Specific disruption of PER homodimers results in drastically impaired behavioral and molecular rhythmicity, pointing the biological importance of this clock protein complex. Analysis of PER subcellular distribution and repressor competence in the PER dimer mutant revealed defects in PER nuclear translocation and a disruption of rhythmic period transcription. The striking similarity of these phenotypes with that of reduced CKII activity suggests that the formation or function of the PER dimer is closely linked to this kinase. Our results confirm a previous structural model for PER and provide strong evidence that PER homodimers are important for circadian clock function.

## Author Summary

The current models of circadian clocks in flies and mammals involve the formation of complexes between clock proteins in the cytoplasm. These complexes are usually heterodimers (that is, made up of two different clock proteins) and appear to enter the nucleus at certain times of the circadian day in order to shut down their own gene expression by deactivating specific transcription factors. After progressive phosphorylation the repressor proteins eventually are degraded so that a new cycle of transcription can begin. Here we present evidence that in addition to heterodimeric complexes, the clock protein PERIOD (PER) also forms homodimers (pairs of identical proteins). Based on a structural model a PER mutant was designed, which is not able to form homodimers but can still bind to its partner TIMELESS (TIM). Flies expressing this mutant PER protein show abnormal clock function in regard to PER nuclear translocation, repressor activity, and behavioral rhythms. The circadian clock model in flies therefore needs to be extended by adding the PER:PER homodimer as a functional unit. Recent structural studies with mammalian PER proteins suggest that homodimers between clock proteins are an important general feature of eukaryotic clocks.

## Abstract 3

The circadian molecular clock model needs to be extended by adding the PERIOD:PERIOD homodimer as a functional unit in rhythm generation in *Drosophila*. Blocking this dimerization leads to faulty nuclear localization, reduced repressor activity, and impaired behavioral rhythms.

## Introduction

Circadian clocks likely evolved because they provide organisms with the advantage to anticipate changes of environmental conditions. Thanks to such clocks, metabolism, physiology, and behavior can be tuned to occur at advantageous times during the 24-h day [1]. Molecularly, circadian clocks are assembled by transcriptional feedback loops in which several clock gene products regulate their own and other clock genes' transcription [2]. In *Drosophila*, the first two clock genes identified were *period* and *timeless*, which are both required for the maintenance of circadian clock function (e.g., [3,4]). The Period (PER) and Timeless (TIM) proteins are able to form a heterodimeric complex, which is important for stabilization of both proteins, for nuclear entry, and presumably also for the function of the PER:TIM complex as transcriptional repressor of their own expression [5–12]. More recently, PER has been shown to enter the nucleus and exhibit repressor activity independent of TIM in vitro and in vivo [13–20]. PER and TIM repress their own expression by binding to their transcriptional activators Clock (CLK) and Cycle (CYC), two b-HLH PAS domain-containing transcription factors that bind E-box sequences in the regulatory regions of *per*, *tim*, and other clock or clock-controlled genes [10,21]. Binding of PER:TIM to the CLK:CYC complex ultimately results in the release of CLK:CYC from the E-boxes of their target genes thereby deactivating them [10].

The kinase encoded by the *double-time* (*dbt*) gene can phosphorylate PER, which results in proteasomal degradation of cytoplasmic and nuclear PER in vivo [22–25]. DBT translocates to the nucleus in a complex with PER (or PER:TIM) [23], and recent work suggests that PER phosphorylation by DBT counteracts PER nuclear entry in vivo [20]. In addition PER may serve as a “bridge” to enable CLK phosphorylation by DBT, which may be a crucial event in inactivating CLK transcriptional activity in cell culture [26–29]. Importantly, the currently available in vivo data do not support this role for DBT. In flies, the lack of PER phosphorylation in a *dbt* mutant background in the absence of TIM is correlated with strong, DBT-independent PER repressor activity [18,20].

In addition to DBT, CKII has also been implicated in phosphorylating PER thereby enhancing PER nuclear entry and repressor activity in vivo [30–33] and in vitro [14]. De-phosphorylation of PER [34] and CLK [27] by the phosphatase PP2A counteracts DBT and CKII mediated phosphorylation, providing an additional level of nuclear translocation and repressor activity regulation. By an apparently independent mechanism, the bHLH-Orange domain transcription factor Clockwork Orange (CWO) also regulates E-box driven expression of clock genes (including *per* and *tim*) by directly binding to CLK target sequences [35–37]. Although it was initially postulated that CWO acts as a repressor, more recent work demonstrates that CWO has also activating properties [38].

In order for PER to exhibit its repressor function, be it direct via altering CLK conformation upon binding, or indirectly by bringing the kinases DBT and CKII into the proximity of CLK, PER needs to be present in the nucleus. Although it had originally been postulated that the PER:TIM interaction is required for nuclear translocation of both proteins, it seems now generally accepted that in flies PER and TIM can enter the nucleus separately [16,39]. These findings were further underscored by the discovery of the *tim^blind^* mutation, which interferes with TIM but not with PER nuclear localization [40]. A Förster Resonance Energy Transfer (FRET)-based study performed with PER and TIM proteins in an embryonic *Drosophila* cell line (S2) revealed that PER and TIM form a complex immediately after their synthesis in the cytoplasm, but separate right before nuclear translocation and enter the nucleus independently [17]. Interestingly, cytoplasmic PER^L^:TIM complex formation is not delayed, but PER^L^ did delay nuclear accumulation [17]. This suggests that events during or after PER:TIM formation are important for the correct timing of nuclear entry. These events most likely involve the reciprocal regulation of the phosphorylation status of PER by DBT and CKII, whereby CKII function seems crucially important for efficient nuclear localization of PER in wild-type flies [20,32,33,41].

**Figure 1:**
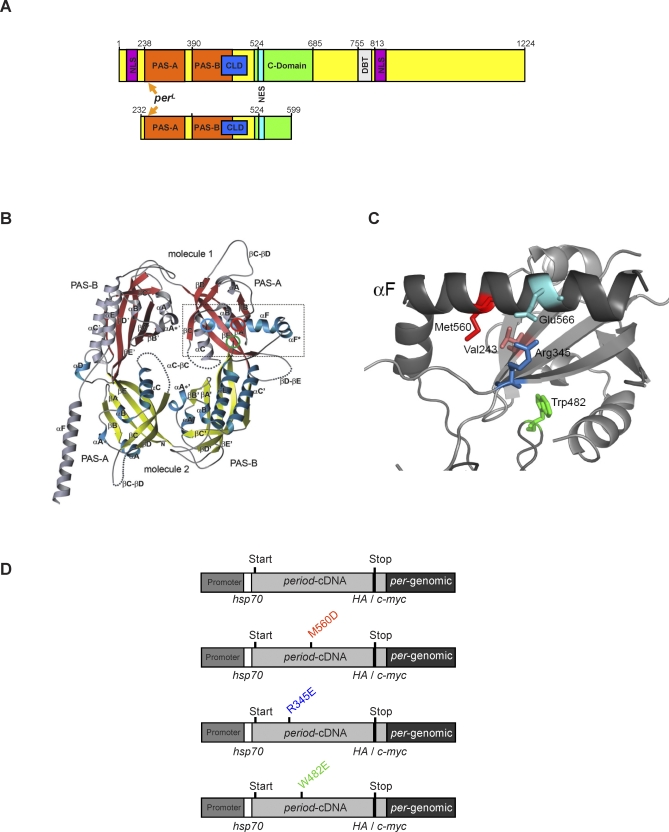
Structure of the PER Protein and Germline Transformation Constructs Encoding Wild-Type and Mutant PER Proteins (A) Cartoons of the PER structure (amino acids 1–1,224) depicting various functional domains identified by in vitro approaches (upper panel) and of the fragment used for crystallization (amino acids 232–599, lower panel). (B) 3-D structure of this fragment (adapted from [44]). The approximate positions of the residues Arg345 (red circle), Trp482 (green circle), and Met560 (blue circle) are indicated. (C) Magnification of the αF:PAS-A interaction surface, where in addition to the mutated residues, the site mutated in *per^L^* is indicated. (D) DNA-constructs encoding wild-type or mutants PER proteins fused to HA or c-MYC tags at their C terminus. Several transgenic lines for each of these constructs where generated (see Materials and Methods). The respective changes are indicated in the same color as in (A). Abbreviations: NLS, nuclear localization domain; PAS, Period-Arnt-Sinlgeminded protein-protein interaction domain; CLD, cytoplasmic localization domain; DBT, Doubletime interaction domain.

In order to enter the nucleus in absence of TIM, PER needs somehow to be protected from DBT-induced degradation. One possible way to stabilize PER in the absence of TIM could be the formation of PER:PER homodimers, which could either form after the PER:TIM complexes dissolve, or co-exist with PER:TIM. The existence of such dimers has long been postulated and even been demonstrated in vitro and in vivo [5,42,43], although they were predicted to exist in very low concentrations [5]. More recently, the crystal structure of a PER fragment (amino acids 232–599) containing the two PAS domains (PAS-A and PAS-B) plus 75 additional C-terminal amino acids has been resolved [44]. It also revealed that this fragment can form a homodimer mediated by several intermolecular interactions between PAS-A, PAS-B, and an α-helix (αF) immediately C-terminal to the PAS domains (Figure 1A–1C). Importantly, one contact is made between Val243 in the PAS-A domain of molecule 1 (the site of the original *per^L^* mutation V243D; [45]) and residues Met560 and Met564 in the αF-helix of molecule 2 (Figure 1B and 1C; [44]). Val243 has previously been associated with mediating PER:PER, PER:TIM, as well as intramolecular PER interactions in vitro [42,43,46]. The long circadian period of *per^L^* flies was attributed to a delayed nuclear entry of the PER^L^ protein, which can be observed in vivo and in vitro [17,47], suggesting that PER:PER and/or PER:TIM interactions regulate nuclear entry time.

Yet, the functional significance of homodimer formation has so far only been tested by analyzing the V243D and M560D PER mutants in vitro [44]. Both amino acid replacements were predicted to weaken the PAS-A:αF interaction by introducing a negative charge into the hydrophobic interface and resulted in increased nuclear translocation and repressor activity of the mutated PER proteins in a cell culture transcription assay [44]. Although this indicated biological relevance for both the PER:PER dimer and the PAS-A:αF interaction, to date no supporting in vivo data exist. Here we show that by weakening the PAS-A:αF interaction via introducing a single amino-acid substitution in αF (M560D) we can drastically reduce PER:PER dimer formation in the fly without compromising the formation of PER:TIM complexes. Moreover, this reduction of homodimer formation coincides with severely impaired behavioral rhythmicity under free running conditions, indicating that the PER:PER dimer is important for clock function. Contrary to the in vitro results described above, our results indicate that PER:PER formation is necessary for efficient nuclear translocation of PER and subsequently for repressing CLK:CYC mediated transcriptional activation.

## Results

### Generation of PER Mutants Predicted to Disrupt PER:PER Homodimer Formation

Several reports indicated the existence of a PER:PER homodimer, although its relevance and biological function in vivo has not been revealed [5,44]. On the basis of the crystal structure determined for a PER:PER N-terminal fragment (Figure 1A–1C; [44]), we designed single amino acid substitutions aimed to destroy the major contact points between the two PER molecules in the context of the full length protein. The mutations were introduced in the *per* cDNA, which was modified to carry either an HA or *c-myc* tag at the C terminus. Expression of the constructs was facilitated by a 1.3-kb fragment of *per*'s 5′-flanking region, a short *hsp70* activation sequence, and a 2-kb *per* 3′-UTR region, known to participate in regulation of normal *per* expression (Materials and Methods; Figure 1D; [48,49]). All constructs were stably integrated into the genome by *P-*element transformation and analyzed in flies that carry the loss-of-function mutation *per^01^* (Materials and Methods, [50]). Since *per^01^* flies do not express endogenous PER protein, the only source of PER in the flies we generated, stems from the various transgenes.

**Figure 2:**
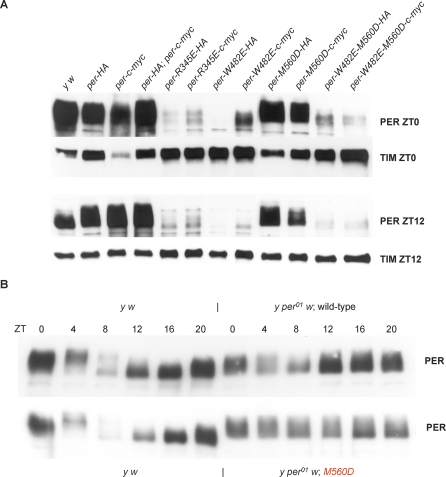
Expression Analysis of Wild-Type and Mutant PER Proteins in Fly Heads Protein extracts from adult fly heads were prepared as described in Materials and Methods at the Zeitgeber Times (ZT) indicated. As *per^+^* control flies the *y w* strain was used. In all other lines PER expression occurred from the transgene indicated above the blot in an otherwise *per^01^* genetic background. Blots were incubated with anti-PER (A, B) and anti-TIM antibodies (A). All transgenes were present in homozygous conditions and the following lines were used (A) *per-HA 1-5-2; per-c-myc 2-2-2; per-HA;per-c-myc* double homozygous made of lines *1-5-2* and *2-2-2; R345E-HA 3–4; R345E-c-myc 4–4; W482E-HA 5–2; W482E-c-myc 6–2; M560D-HA 9-4-2; M560D-c-myc 10-2-2; W482E-M560D-HA 11–6; W482E-M560D-c-myc 12–1.* (B) *per-c-myc 2-2-2; M560D-HA 9-4-2*. The low PER and TIM signals in the *per-c-myc* lane at ZT0 are caused by partial degradation of the protein sample.

As controls, we first generated the wild-type versions of the tagged *per* constructs (Figure 1D) and tested the PER expression levels on western blots at ZT0 and ZT12 (reflecting times of high and low PER expression levels in wild-type flies, respectively). We found robust expression of both HA and *c-myc* tagged versions when we compared the transgenes to *per^+^* control flies (Figure 2A). We next created a mutant PER version, in which the Trp at position 482 was exchanged to Glu, predicted to disrupt the two (reciprocal) interaction points between PAS-B and the PAS-A domain of the dimerizing molecule (Figure 1B–1D). Western-blot analysis of several independent W482E transgenic lines revealed that they accumulate only very little W482E PER protein compared to the *per^+^* and wild-type transgenic controls (Figure 2A). We obtained the same results when the blots were incubated with anti-HA antibodies, indicating that the low anti-PER signals are not due to altered PER-immunoreactivity of the mutant proteins. Quantitative analysis of mRNA expression driven from the W482E construct indicates that reduced mRNA levels do not account for reduced protein levels (Figure S1). The most likely explanation for reduced W482E levels is therefore reduced protein stability (see Discussion).

We next introduced a missense mutation at position Arg345 of PER located in the PAS-A domain. The R345E mutation we introduced is expected to disrupt the salt-bridge observed between Arg345 in PAS-A of molecule 1 and Glu566 in the αF-helix of molecule 2 (Figure 1B–1D); [44]). As in the case of W482E, flies expressing the R345E transgene accumulated only very little R345E PER compared to controls, indicating that this mutant protein is also unstable (Figure 2A and Figure S1, see Discussion).

R345E and W482E carry a mutation in the PAS-A, and CLD domain, respectively, which have been described to be involved in binding to the clock protein TIM in cell culture [11,46]. Therefore, instead or in addition to disrupting PER:PER binding, the mutations could interfere with formation of the PER:TIM dimer. Since PER and TIM heterodimerization is thought to stabilize PER [5,7], this could explain the low levels of our mutant PER proteins. We therefore decided to generate a PER mutant that, based on the 3-D structure, should interfere only with the formation of PER:PER dimers and not with the PER:TIM interaction. Residue Met560 is situated in the αF-helix contacting Val243 in the PAS-A domain of the dimerizing molecule (Figure 1B–1D). Although Val243 has been implicated both in PER:PER and PER:TIM interactions (it is the site of the original *per^L^* mutation: V243D), no such interactions have been reported for any residues in the αF-helix or C-domain. The M560D mutant we generated indeed exhibited robust levels of mutant PER protein, indicating that stabilizing PER:TIM interactions are not disrupted by the mutant (Figure 2A). In agreement with the results just described, a PER W482E-M560D double mutant expressed a low level of mutated PER protein (Figure 2A and Figure S1).

Since the M560D mutant exhibited wild-type levels of PER, we analyzed the temporal expression profile of this mutant PER protein. For this, we performed western blots with extracts prepared from flies at six different time points throughout the day, which were probed with anti-PER antibodies. We compared temporal expression between nontransgenic *per^+^* control flies, PER wild-type transgenics, and the M560D mutants at six different time points throughout the day (Figure 2B). The nontransgenic *per^+^* controls exhibited the typical robust PER oscillations in abundance and mobility (because of temporal regulated phosphorylation, [51]) (Figure 2B, left panels; e.g., [52]). Similarly, the transgene encoded wild-type PER proteins exhibited daily abundance and mobility oscillations, although the mobility shifts appeared to be reduced (Figure 2B, upper right panel). The reduced mobility changes observed for the transgene-encoded wild-type proteins were apparent in several independent transgenic lines both containing the HA or *c-myc* tag (unpublished data). Since the transgenic proteins contain the entire PER open reading frame, the altered migration properties are likely caused by the different tags attached to the C termini of PER.

Nevertheless the wild-type transgenic PER proteins underwent robust daily oscillations, which were absent or severely diminished from the M560D mutant proteins (Figure 2B and Figure S2A). The M560D protein was almost equally expressed during the 24-h day; and during all time points analyzed, fast and slow migrating mutant PER species were present. Quantification of the band intensities revealed that fast migrating, hypophosphorylated forms of PER are more abundant in the late night in the M560D mutant compared to the slower migrating phosphorylated forms (Figure S2B and Figure 2B; note the distinct fast migrating forms in the mutant at ZT16 and ZT20). Although as discussed above, the tags attached to the proteins likely contribute to this effect, this finding indicates that the M560D protein is less efficiently phosphorylated compared to the wild-type protein. In any case, a significant reduction in the overall PER abundance rhythm could be observed in all M560D transgenic lines analyzed, independent of the insertion site or type of tag (Figure 2B and Figure S2). If the M560D mutant indeed interferes with PER:PER homodimerization, our results imply that dimer formation is necessary for proper PER cycling. We therefore tested whether wild-type polypeptides form homodimers in the living fly, and if their formation may be compromised by the M560D mutation.

### PER:PER Dimers Are Present in the Fly and Are Disrupted by the M560D Mutation

**Figure 3:**
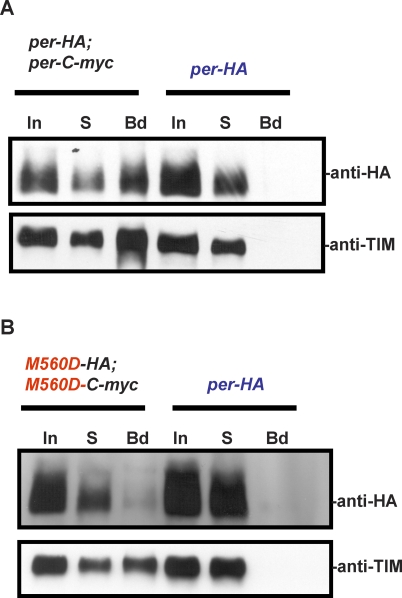
Formation of PER:PER Dimers Is Disrupted by the M560D Mutant CoIP experiments with flies containing double-homozygous wild-type PER (A) or M560D PER (B) encoding transgenes fused to HA and *c-myc* tags (lanes 1–3), or homozygous transgenes fused to HA (lanes 4–6). CoIPs were performed with flies collected at ZT20 as described in Materials and Methods. Western blots were incubated with anti-HA and anti-TIM antibodies as indicated. Protein extracts of flies expressing both an HA and *c-myc* (lane 1), or only the HA tagged version of PER (lane 4) were loaded as Input control (In). In lanes 3 and 6, proteins eluted from anti-c-MYC coated beads are loaded (Bd) for double tagged, and singly HA tagged flies. Lanes 2 and 5, Supernatant (S) after binding the extracts to anti-MYC coated beads. The strong anti-HA signal in lane 3 (upper panel) clearly indicates binding of HA-tagged PER to MYC-tagged PER (i.e., homodimer formation), whereas the weak signal in the lower panel of lane 3 indicates severe disruption of homodimer formation. The lack of signal in lane 6 indicates that HA-tagged proteins do not bind unspecifically to anti-MYC beads. Note that both wild-type and M560D mutant proteins bind to TIM (lane 3, upper and lower panels). This experiment was repeated three times with similar results, except that the anti-HA signal of M560D flies was completely absent in the “Beads” fraction in the other two trials (see also Figure 5A).

Homodimer formation in flies was tested by performing co-immunoprecipitation (CoIP) experiments making use of the HA and *c-myc* tags attached to the wild-type and mutant PER proteins. *per^01^* flies homozygous (on Chromosome *2*) for a transgene encoding a HA-tagged PER and homozygous (on Chromosome *3*) for a transgene encoding a *c-myc*-tagged PER were synchronized to a 12-h:12-h light–dark (LD) cycle and collected at ZT20, a time where homodimer formation was previously reported [5]. Protein head extracts were subjected to CoIP, whereby the extracts were incubated with anti-*c-myc* coated sepharose beads and the western blots were incubated with anti-HA antibodies (Materials and Methods). Therefore, HA-tagged PER proteins can be detected on the western blot only when they had formed a homodimer with the *c-myc*-tagged PER molecules. In agreement with the earlier study [5], we were able to detect PER-HA, indicating PER:PER homodimer formation at ZT20 (Figure 3A, lane 3). To test if HA-tagged PER molecules are able to bind to anti-c-myc coated beads unspecifically, we subjected extract from flies expressing only the PER-HA proteins to the same CoIP. The lack of anti-HA signal (Figure 3A, lane 6) clearly shows that PER-HA can only be pulled down in the presence of PER-c-myc. It should be mentioned that we are only able to detect homodimers formed between HA and c-myc tagged molecules and not PER-HA:PER-HA and PER-c-myc:PER-c-myc dimers, which are likely to be formed at similar rates. Therefore, the overall levels of PER:PER homodimers are expected to be two times higher compared to those we detected in our CoIP experiments (there are two possible ways to form PER-HA:PER-c-myc dimers, but only one to form dimers between PER molecules carrying identical tags).

The same experiment was then performed using double-homozygous *per^01^* M560D-HA/M560D-*c-myc* flies. In contrast to the wild-type tagged proteins, M560D mutant PER proteins were not able to support homodimer formation, since little to no PER-HA signals could be detected (Figure 3B, lane 3). But given the fact that we can detect only 50% of the possible PER:PER dimers (see above) we cannot rule out that some homodimers form between the mutant PER M560D proteins.

Given that M560D proteins are stable we assumed that they are still able to heterodimerize with the TIM protein (see above). To test this idea, we also incubated the western blots after CoIP with anti-TIM antibodies. Indeed, both the wild-type PER and the mutant M560D proteins strongly bound to TIM (Figure 3A and 3B, lanes 3). This therefore explains the stability of the mutant M560D protein. More important, since M560D-PER:TIM heterodimerization is not affected, the M560D mutant allows for specific functional analysis of PER homodimerization owing to absent or drastically reduced PER:PER formation.

### The M560D Mutant Interferes with Normal Behavioral Rhythmicity

**Figure 4:**
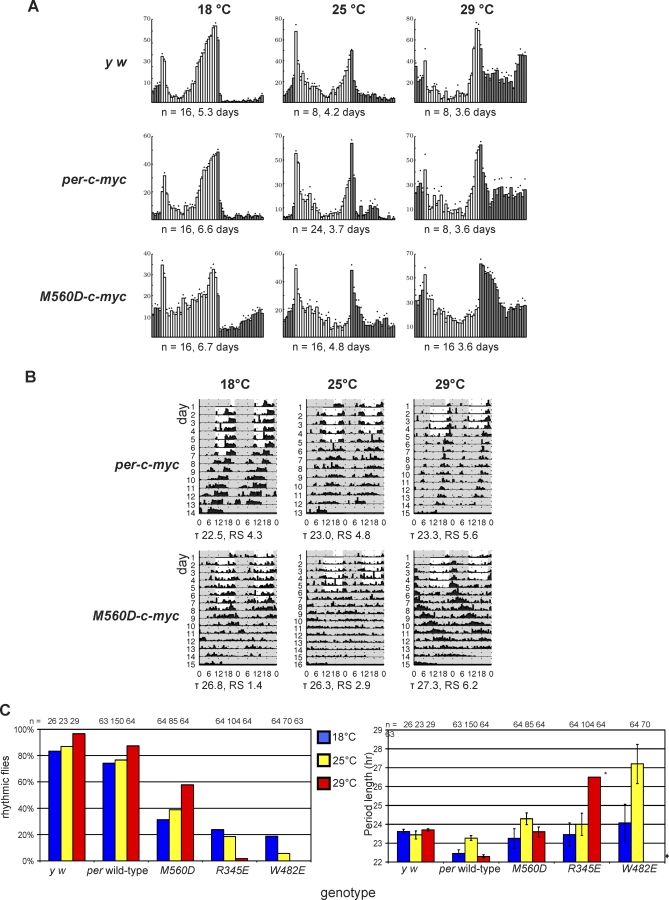
PER:PER Dimer Formation Is Important for Normal Locomotor Rhythms Locomotor rhythms of nontransgenic *per^+^* control flies (*y w*), *per^01^* flies carrying the wild-type PER encoding transgene, or the M560D encoding transgene (both fused to either HA or c-MYC). Flies were kept for 4–7 d in LD followed an additional week in DD at the temperatures indicated above each panel. (A) Daily average plots of control and mutant flies for the LD portion of the experiment. Note that M560D mutants show less robust anticipation of the LD and DL transitions, but are still able to shift their activity to the light or dark portion in cold and warm temperatures, respectively. For *per-c-myc* data from 4 transgenic lines were pooled (2-2-1, 2-2-2, 2–3, and 2–6). For *M560D-c-myc* data from lines 10-2-1 and 10-2-2 were pooled. (B) Individual actograms of the LD and DD part of the experiment showing control (line 2–6) and mutant flies (line 10-2-2). For the M560D mutant long-period rhythmic individuals were selected to demonstrate that the period remains almost constant at the different temperatures (i.e., normal temperature compensation). Similar results as shown in (A) and (B) were obtained with the respective *HA-*tagged lines or lines expressing both transgenic types (unpublished data, [C] and Table S1). (C) Quantification of DD behavior in control and mutant M560D, R345E, and W482E flies. Shown are averages of all transgenic lines analyzed (Table S1). M560D mutants show a drastic reduction of rhythmicity compared to controls, whereas the other mutants are almost completely arrhythmic (left panel). Temperature compensation is normal in controls and M560D mutants but impaired in the few rhythmic individuals of the other 2 mutants. *, only one rhythmic fly; ♦, all flies were arrhythmic; n, the number of animals included in the calculation.

To determine if the PER homodimer fulfills biological function we analyzed locomotor activity rhythms of wild-type and mutant PER-encoding constructs in a *per^01^* genetic background. Behavior was analyzed in 12-h:12-h LD cycles and in constant darkness (DD) to assess effects on synchronization to LD cycles and on the internal clock. The M560D mutation is predicted to disrupt the interaction with Val243, the site of *per^L^* mutation, which is defective in temperature compensation [53]; therefore we tested LD and DD behavior at different constant temperatures of 18 °C, 25 °C, and 29 °C. As expected, the wild-type PER-encoding constructs were able to restore robust and largely temperature compensated behavioral rhythms in *per^01^* flies, both in LD and DD conditions (Figure 4 and Table S1). In LD at 25 °C, *per^+^* control and wild-type transgenic flies showed the characteristic anticipation of the D to L and L to D transitions in the mornings and evenings, respectively interspersed by prolonged periods of inactivity during the day (siesta) and night (Figure 4A, cf. [54]). Moreover, at cold (18 °C) and warm (29 °C) temperatures, flies moved their activity peaks to occur mainly in the light and dark portions of the day, respectively (cf. [55]).

Next, we analyzed behavior of the M560D mutant flies. Although they behaved similarly to the controls overall, behavioral anticipation of the environmental changes was less pronounced. For example at 25 °C, when wild-type control flies where relatively inactive during the siesta, M560D mutant flies showed increased activity levels (Figure 4A). On the other hand, M560D mutants were still able to move their periods of main activity towards the light or dark phase in cold and warm temperatures, respectively (Figure 4A).

In DD and 25 °C about 80% of the *per^01^* flies transformed with the wild-type PER-HA or PER-*c-myc* encoding constructs exhibited robust circadian rhythms with periods of ∼23 h (Figure 4B and 4C). Moreover, the number of rhythmic animals and the period length did not vary significantly at 18 °C and 29 °C, indicating that the PER-HA and PER-*c-myc* fusion proteins are able to replace endogenous PER protein (Figure 4B and 4C and Table S1). In contrast, depending on the temperature, only between 30% and 60% (40% at 25 °C) of the M560D mutant flies were rhythmic in DD, indicating that the circadian clock is drastically impaired in these flies (Figure 4C and Table S1). Nevertheless, the period length did not vary much between the different temperatures, indicating that temperature compensation is not affected (Table S1). As was observed for flies from the two control strains, overall rhythmicity for M560D flies was correlated with an increase in temperature (Figure 4C, left panel). At each of the different temperatures tested the M560D mutants showed slightly longer periods compared to transgenic controls (∼1 h longer at each temperature, Figure 4C, right panel; Table S1). This period lengthening could be due to the specific mutation, since in the homodimer M560D is predicted to disrupt the interaction with the *per^L^* site Val243. Alternatively, slightly reduced overall PER levels in the M560D transgenics compared to the wild-type transgenics could account for the period lengthening, because *per* function is dosage sensitive (e.g., females carrying only one copy of the *X-*linked *per* ^+^ allele have 25-h periods, [50]). In any case, together with the biochemical results described, the behavioral defects observed in the M560D mutants clearly point to a crucial function of the PER:PER dimer within the circadian clock.

We also analyzed the behavioral rhythms of the other mutants we generated (W482E, R345E, and the double mutant W482E M560D). Neither of the mutant proteins was able to restore rhythmic behavior in *per^01^* in LD or DD conditions in more than 20% of the flies (Figure 4C and Table S1), consistent with the low mutant PER protein levels present in these flies. The few rhythmic flies exhibited widely variable periods, and temperature compensation was severely compromised (Figure 4C and Table S1). Although this effect can be due to many different reasons, it may indicate that PER:TIM interactions are more important for proper temperature compensation compared to PER:PER (temperature compensation in M560D flies is normal, Figure 4C).

### The M560D Mutant Reduces the Repressive Function of PER In Vivo

**Figure 5:**
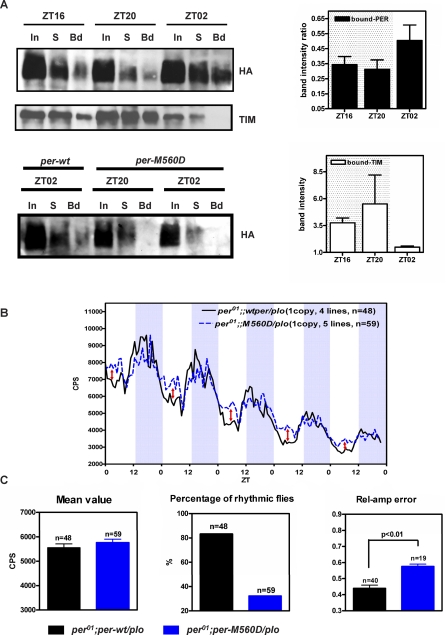
The PER:PER Dimer Is Important for PER Repressor Activity (A) Temporal profile of PER:PER and PER:TIM dimer formation at three different time points at 25 °C was analyzed by CoIP (see legend to Figure 3 and Materials and Methods for details). Three independent experiments were performed. “Band intensity ratio” indicates the relative amount of PER homodimers. It represents the ratio between the HA signals in the “Bd” fraction and that in the “In” fraction. For TIM, band intensities where averaged. At ZT2 significant more PER:PER dimers are formed compared to ZT16 and ZT20. (B) Transcriptional rhythms of *per* expression were measured in vivo using the real-time *luciferase* measurements in adult flies of the *plo* transgenic type in a *per^01^* genetic background (Materials and Methods). An average of four *per-*wild-type transgenics (both HA and c-MYC tagged) shows clear rescue of rhythmic *plo* expression (usually expression of this construct is arrhythmic in a *per^01^* genetic background [56,57]). In contrast the M560D mutant results in severely dampened transcriptional *per* rhythms (blue line) and less-pronounced trough levels of *plo* expression, indicating faulty repressor activity mediated by the M560D PER. (C) Quantification of expressions levels (left) and determination of the significance of rhythmicity for each time series by FFT-NLLS analysis. M560D drastically reduces rhythmicity (middle) and the few rhythmic flies show significantly increased rel-amp errors, indicative of weak rhythmicity (Material and Methods).

So far our data indicate that disruption of the PER:PER dimer interferes with circadian clock function and results in abnormal behavioral rhythms (Figures 2B, 3, and 4). But what specific clock process(es) would be carried out by PER:PER dimers? One possibility we tested is that PER's function as a repressor of CLK/CYC activated *per* and *tim* transcription involves the PER:PER dimer. In order to test this we first analyzed if the abundance of PER:PER dimers varies throughout the circadian day. If the PER:PER homodimer is important for PER's function as a repressor, one would expect to see a higher accumulation of the dimer at times when PER is nuclear, which corresponds roughly with the second half of the night until the early day (ZT18 to ZT4) [16,47]. We therefore performed CoIP experiments with our PER-HA and PER-*c-myc* expressing *per^01^* flies collected at ZT16 (cytoplasmic) and ZT20 (nuclear). We also included an early morning time point (ZT2), in which PER is in the nucleus and TIM is mostly degraded due to the presence of light [5]. During this time PER has been shown to be the main repressor of CLK/CYC induced transcription [13], and perhaps PER:PER homodimer formation is crucial for this TIM-independent function of PER. Indeed, the temporal analysis of PER:PER abundance revealed that peak amounts are reached at ZT2, suggesting that the PER:PER dimer is an active repressor unit (Figure 5A). Consistent with this idea, PER:PER homodimer formation in the M560D mutant is disrupted at the two nuclear time points (ZT20 and ZT2) investigated (Figures 3B and 5A, lower panel). But also at times when PER is cytoplasmic (ZT16), substantial amounts of PER:PER complexes exist, similar to what is observed when PER is nuclear (ZT20), suggesting a role for PER:PER during the accumulation phase of this clock protein (Figure 5A).

These results suggest that the homodimer acts as repressor. In order to further test this hypothesis we wondered if the M560D mutant would decrease PER's repressive activity. For this, we made use of a *period-luciferase* (*per-luc*) transgenic reporter strain that reflects *per* transcription in vivo [56,57]. In the *plo* transgene the *per* promoter is directly fused to the firefly *luciferase* cDNA and *per-luc* expression in individual adult flies can be monitored with an automated bioluminescence counter (e.g., [57]). As expected, the wild-type PER constructs restored robust transcriptional rhythms when introduced into *per^01^ plo* flies (Figure 5B and Table S2). Interestingly, when the same reporter flies expressed the M560D PER mutant, transcriptional rhythms were abolished in the majority of the flies, or they were of significantly reduced amplitude (Figure 5B and 5C and Table S2). Although the overall mean levels of *plo* expression were similar between the wild-type and mutant flies, the latter did not reach the trough levels of expression observed in the wild-type PER transformants; or if they did, only for a very limited amount of time (Figure 5B, arrows). We conclude that repression by the mutant monomeric PER protein is less efficient compared to that of the dimeric wild-type protein, resulting in a breakdown of *per* transcriptional rhythms.

### The M560D Mutant Interferes with Nuclear Translocation of PER

**Figure 6:**
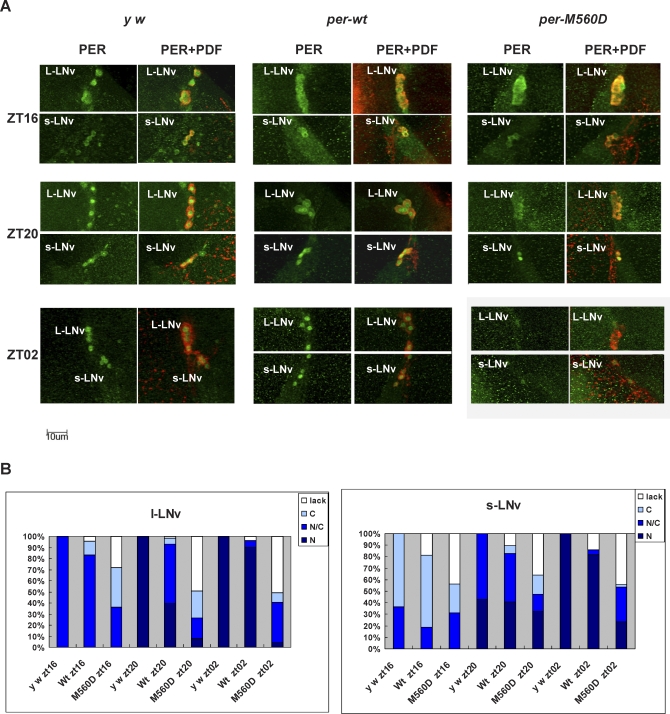
The M560D Mutation Interferes with Nuclear Translocation of PER (A)Whole mounted brains prepared from control and M560D flies were prepared from flies collected at the indicated ZT times at 25 °C. Anti-PER (green) and anti-PDF (red) stainings (Materials and Methods) revealed normal cytoplasmic and nuclear localization in the large and small LNv's of nontransgenic control flies (left panel) as well as *per^01^ per-*wild-type transgenics (middle panel). The M560D mutant exhibits overall less PER-positive LNs, and in the remaining neurons nuclear translocation is severely impaired (see Results for details). (B) Quantification of staining results separate for small and large LNvs (see Materials and Methods for details). Lack, neurons without any detectable PER staining; C, N, neurons with predominantly cytoplasmic or nuclear signal, respectively; N/C, equal staining intensity in both compartments. The transgenic lines used for this analysis were the same than the ones for the CoIP analysis. For each genotype the following numbers of brain hemispheres (*n*) were analyzed: *y w* (ZT16, *n* = 8; ZT20, *n* = 7; ZT2, *n* = 5; M560D [ZT16, *n* = 13; ZT20, *n* = 15; ZT2, *n* = 12), *per* wild-type (ZT16, *n* = 12; ZT20, *n* = 14; ZT2, *n* = 13).

Faulty nuclear translocation could be one possibility why the dimerization defective M560D mutant exhibits reduced repressor activity. Therefore we determined the subcellular distribution of PER within the clock neurons of the adult brain at different times within a circadian cycle. Rhythmic expression and proper cytoplasmic/nuclear shuttling of clock proteins (including PER and TIM) in the lateral clock neurons (LNs) is required for proper clock function and control of rhythmic locomotor activity (e.g., [58]). We stained brains prepared at ZT16 (PER cytoplasmic), ZT20 and ZT2 (both PER nuclear) with anti-PER and anti-PDF (as a marker for cytoplasmic staining in the LNv's; the more ventrally located subgroup of the LNs [58]). In nontransgenic *per^+^* control flies (*y w*) we observed the characteristic cytoplasmic and cytoplasmic plus nuclear staining at ZT16 and predominantly nuclear staining at ZT20 and ZT2 in all clock-neuronal cell types (Figure 6A, left row; Figure 6B). The *per^01^* wild-type transgenics showed a very similar pattern, although the anti-PER signals appeared overall weaker (Figure 6A, middle row; Figure 6B). This is in agreement with the robust behavioral rescue of *per^01^* mediated by these transgenes (Figure 4 and Table S1). When we performed the same stainings in the *per^01^* M560D mutant flies, we detected a significant reduction of the total numbers of LNs expressing PER (Figure 6A and 6B, and Figure S3). In the PER positive cells at ZT16 a significant (*p* < 0.05) reduction of cytoplasmic or nuclear/cytoplasmic signals was visible in the s-LNv and l-LNv, respectively (Figure 6A and 6B, and Figure S4). At ZT20 a reduction of cells with nuclear and nuclear/cytoplasmic signals was observed for both cell types (Figure 6A and 6B), but the difference to the wild-type transgenics was significant only for the l-LNv (Figure S4). Importantly, some mutant s-LNv cells exhibited clear nuclear PER signals (Figure 6A and 6B), indicating that the results can not simply be the cause of overall lower PER levels in the mutant. At ZT2, when nuclear PER accumulation is maximal in the nontransgenic and transgenic controls (Figure 6A and 6B), for both cell types we observed significantly fewer cells with nuclear signals, paralleled by an increased number of cells with weak nuclear and cytoplasmic signals (Figure 6A and 6B, and Figure S4). This clearly points to impaired nuclear localization efficiency in the M560D mutant flies in both s-LNv and l-LNv. This abnormal nuclear-cytoplasmic distribution is in good agreement with the poor behavioral rescue and with the reduced repressor activity, mediated by this mutant protein (Figures 4 and 5B, and Table S1). These results further point to the importance for PER:PER homodimer formation for circadian clock function and to a role for the dimer in PER nuclear localization and transcriptional repression.

## Discussion

We present here the first evidence, to our knowledge, for in vivo function of a PER:PER homodimer. A single amino-acid replacement (M560D) largely disrupted homodimer formation, resulting in severe behavioral and molecular phenotypes. These results point to a prominent function for the PER dimer within the circadian clock. The mutation just noted specifically interferes with PER:PER and not PER:TIM formation, indicating that the observed phenotypes are due to faulty PER:PER dimer formation. The normal interaction between the mutant PER protein and TIM also indicates that the circadian phenotypes are not due to abnormal function of a monomeric PER protein, although we can not completely rule out this possibility. Moreover, this alternative interpretation of our data would imply that formation of PER:PER dimers (which occurs within the fly and is probably under circadian control: Figures 3A and 5A) has no functional meaning, because disruption of this homodimer would have no effect on the clock. The most likely interpretation of our data is therefore that the effects caused by the M560D mutation are due to the disruption of the PER:PER homodimer rather than being caused by a faulty monomeric PER protein.

### Residues Important for PER:PER and PER:TIM Interactions

Our in vivo findings are supported by biochemical studies analyzing homodimer-formation of the N-terminal crystallized PER:PER fragment (amino acids 232–599). Although the M560D mutant in the context of this fragment runs as a dimer in gel filtration experiments, the affinity of the dimer is significantly reduced by the mutation (see accompanying report). Furthermore, the V243D mutant (*per^L^*) version of this PER fragment and a fragment entirely lacking the αF-helix behave as monomers in gel filtration, demonstrating the importance of the PAS-A-αF interface (including Val243 and Met560) for dimerization in solution [44].

Unlike our in vivo results, when the M560D mutant in the context of the whole PER protein was expressed in S2 cells, it efficiently entered the nucleus and also acted as a potent repressor [44]. This discrepancy is likely linked to the fact that events in S2 cells not necessarily reflect the in vivo situation (for example TIM was not co-expressed in the study just cited) as was observed repeatedly in the past (e.g., cytoplasmic localization of PERΔ in S2 cells versus nuclear localization in flies [28,29]) and underscores the importance of in vivo studies.

Except for the M560D mutation all other mutant proteins we analyzed were unstable in flies. The W482E mutation (Figure 1B and 1C) is predicted to disrupt dimer formation at two symmetrical positions between the Trp482 located at the tip of the βD′-βE′ loop in PAS-B and the hydrophobic pocket formed by the βA, αB, and αC strands and helices of the other PER molecule [44]. As expected from this disruption of a prominent dual interaction point, the W482E weakens the dimer in the context of the PER (232–599) PAS domain fragment (see accompanying Research Article). Moreover, and similar to M560D, in S2 cells a W482A mutation shows enhanced repression compared to the wild-type fragment in the context of full length PER [44]. Since the purified PAS domain fragment carrying the W482E mutation is stable and properly folded (see accompanying Research Article), the instability of the W482E mutation in flies suggests that in vivo additional factors contribute to PER stability. One important feature missing from the stable PAS domain fragments, is a newly discovered N-terminal interaction domain with SLIMB, which is required for efficient degradation of PER [24]. The same applies for the R345E mutant, which is expected to disrupt a salt bridge between Arg345 on the βD strand of molecule 1 and Glu566 located on the αF-helix of molecule 2 (Figure 1B, 1C). Most likely the TIM protein and phosphatase activity supply this additional stability in vivo as discussed above. All mutants analyzed in the current study (except M560D), map to the PAS-A (R345E) or the PAS-B (W482E) domains, which have been implicated in directly mediating the PER:TIM interaction ([11,46], also see accompanying report). The most simple explanation for the observed instability of PER mutants containing amino acid changes in one of the PAS domains is therefore that they also interfere with the PER:TIM interaction (see accompanying Research Article). If true, mutants mapping to the αF helix and predicted to weaken dimer formation (like M560D) should result in stable proteins that are still able to interact with TIM. Such candidates include Met564 and Glu566, predicted to weaken the interaction with Val243 and Arg345, respectively [44].

The *per^L^* mutation (V243D) lengthens the free-running period dramatically [50] and also compromises temperature compensation [53]. Molecularly, *per^L^* leads to a temperature-sensitive delayed nuclear entry of PER^L^, which can account for the long behavioral period and loss of temperature compensation in this mutant [47]. Structural and in vitro studies [42–44] suggested an important role for Val243 in PER homodimerization. In agreement with these studies, we revealed that the contact amino acid of Val243 in the αF helix of the partner PER molecule (Met560) is also crucial for homodimerization in the context of the full length PER protein in vivo. But in contrast to the original and in vitro mutagenized *per^L^* mutants [42,47,53], our M560D mutant flies only exhibit a slight (1-h) increase in period length and no loss of temperature compensation (Figure 4C and Table S1). Therefore we believe that the period and temperature compensation defects of *per^L^* mutants are due to a faulty interaction with TIM as originally proposed by Gekakis et al. [46]. Indeed, we observed normal interactions between M560D PER and TIM, further supporting this hypothesis. It follows, that whereas the Val243 residue of PER is involved in mediating both PER:TIM and PER:PER interactions, the Met560 residue mediates only (or mainly) homodimerization. Further support for the importance of the Val243 residue for PER:TIM interactions came from a genetic screen, in which a “suppressor of *per^L^*” mutation (*tim^SL^*), which ameliorated both phenotypes (period length and temperature compensation), was isolated and found to map to the *timeless* gene [59,60]. Also, *cry^b^* suppresses the temperature compensation phenotype of *per^L^*, indicating that the residue mutated in *tim^SL^* (Thr494) interferes with the CRY:TIM interaction, and that the V243D (*per^L^*) induced temperature compensation defect is caused by increased PER^L^:TIM:CRY interactions [61]. Similar to the model original proposed by Rosbash and colleagues [42], it is therefore possible that the PAS-A:αF interaction between two PER molecules competes with a heterologous PAS-A:TIM interaction, which is likely also influenced by CRY.

### The Biological Function of PER:PER

It has been shown in vitro that, after formation of PER:TIM complexes in the cytoplasm of S2 cells, these dimers dissolve, and both PER and TIM enter the nucleus independently [17]. Also Shafer et al. [16,39] demonstrated advanced nuclear entry of PER (without TIM) in vivo. Therefore, it is possible that PER:PER formation is necessary for, or promotes, nuclear entry after the PER:TIM dimers dissolve.

What could be the possible signal for this event? Two kinases have been implicated in nuclear localization of PER. DBT promotes cytoplasmic localization of PER [18,20,41], perhaps via the DBT-dependent phosphorylation of a PER NLS sequence [24]. CKII supports PER nuclear translocation [33], perhaps because PER (or TIM [62]) phosphorylation by CKII serves as a signal for PER:TIM break down, whereupon PER:PER dimers form that could then enter the nucleus. Alternatively, a different signal could promote the PER:TIM break down, and the PER:PER dimer could serve as a prime substrate for CKII, followed by nuclear translocation. This hypothesis is supported by the fact that CKII mutants and M560D PER mutants share similar phenotypes. Both result in period lengthening without compromising temperature compensation, although CKII mutants have a much more drastic effect on clock speed compared to M560D (Figure 4 and Table S1) [30–33]. Also, in both cases PER oscillations (as determined by western blots) are affected and show a reduction of PER phosphorylation (Figure 2B and Figure S2) [30–33]. Finally nuclear translocation is similarly impaired by M560D and CKII mutants (Figure 6 and Figure S4) [30–33]. Probably as a result of this faulty nuclear localization both mutants exhibit reduced repressor activity of PER (Figure 5B, 5C; [33]), which is in agreement with the enhancing effect of CKII on PER repressor activity observed in cell culture [14]. Both CKII [33] and M560D mutants do not completely block PER nuclear entry, indicating that other factors also support PER nuclear translocation, or that to some extent PER monomers or PER:TIM heterodimers are able to enter the nucleus. It is likely that M560D does not completely block homodimer formation, as indicated by the weak band we sometimes see in the M560D CoIP experiments (Figure 3B). We do see nuclear M560D PER, mainly within the small LNv cells, but much less frequently in the large LNv's. Perhaps this difference is simply due to the smaller cytoplasmic volume in the s-LNv compared to the l-LNv, which could enhance homodimer formation because of a higher local concentration of monomeric proteins. These homodimers would then be able to enter the nucleus to repress CLK. The occasional nuclear staining in the s-LNv cells also explains why not all the M560D mutant flies exhibit arrhythmic behavior in constant darkness. The s-LNv's are crucial for maintaining sustained locomotor rhythms (e.g., [63]), and it has been shown that only a few of these cells are sufficient to drive behavioral rhythms if neuritis projecting from them terminate within the dorsal brain [64].

The small fraction of nuclear PER homodimers presumably also explains that some repression is still maintained in the M560D mutants (Figure 5B). Alternatively, homodimer formation may not be required for repression, which could be mediated by PER:TIM heterodimers during the late night [10], followed by PER repression in the early morning [13]. Given that the M560D mutation leads to less efficient transcriptional repression (Figure 5B), and robust levels of wild-type homodimers are present in the early morning (Figure 5A), we favor the idea that PER does form nuclear homodimers to mediate repression at this time of day.

In support of this hypothesis, a mutant PER protein lacking a rather large piece of the C-domain (ΔC2, missing amino acids 512–568 and therefore the complete αF-helix; Figure 1A) including M560D, shows drastically reduced repressor activity in vivo [65]. In contrast to M560D PER, the ΔC2 protein is constitutively nuclear (unpublished data in [65]), suggesting that the predicted inability of this protein to form homodimers is the reason for lacking repressor activity. The constant nuclear localization of ΔC2 PER in contrast to M560D is possibly due to a potential nuclear export signal located in the deleted region of ΔC2 (Figure 1A [66,67]). The ΔC2 PER is also stable in constant light [65], indicating that, in addition to PER:PER, the previously documented PER:CRY interaction in yeast [68], is also mediated by this domain in vivo (although, see [69]). Alternatively, the large 56 amino acid deletion in ΔC2 PER could result in a structural change of the PER:TIM complex, which may interfere with the CRY:TIM interaction.

DBT promotes PER phosphorylation and turnover, when PER is free from TIM in the cytoplasm and the nucleus [22,23], presumably by creating an optimized binding site for the F-box protein SLIMB [24]. PER can be stabilized either by binding to TIM [7], by preventing progressive DBT-dependent phosphorylation [70] or by phosphatase activity [24,34,71]. Perhaps dimer formation also contributes towards PER stability, although we think this is unlikely because we do observe robust PER levels in the M560D mutants (Figures 2, 3B, and 5A). To obtain a definite answer, mutations that completely disrupt dimer formation without compromising other PER protein interactions need to be generated (see “Residues Important for PER:PER and PER:TIM Interactions” in “Discussion” below).

In principle it is possible that PER:PER complexes bind to DBT, since an important DBT-binding domain has been mapped to a small region (27 to 54 amino acids, depending on the study) located C-terminal of the PAS and αF interaction surfaces (Figure 1A, [28,29]). The DBT-binding domain also overlaps with the previously identified “CLK CYC Inhibition Domain” (CCID), which presumably explains the reduced in vitro repressor activity of PER molecules lacking the CCID [15]. Therefore DBT could enter the nucleus in a complex with PER:PER and mediate transcriptional repression via phosphorylation of CLK, as recently proposed [26–29]. Importantly, flies expressing a PER protein lacking the DBT-interaction domain (PERΔ) accumulate constantly hypophosphorylated forms of nuclear PER [28,29], reiterating that DBT supports cytoplasmic localization of PER and is not required for nuclear entry in vivo [20,41]. Although it was reported that PERΔ can act as potent repressor in cultured “nonclock” cells [29], this deficient protein shows drastically reduced repressor activity in vivo, supporting a model in which action of DBT within the nucleus contributes to repression of CLK [28]. Our findings suggest that, at least in the early morning, after light-dependent clearance of TIM, the PER-PER homodimer is building the proposed “bridge” for CLK phosphorylation by DBT [26,28]. In this model, disruption of the PER:PER dimer mainly affects nuclear accumulation of PER (perhaps because of an impaired interaction with CKII as discussed above); but since DBT interaction is most likely not disrupted, mutant “escaper” complexes that are able to enter the nucleus can still mediate repression. However, it has been shown that in the absence of TIM and DBT activity, PER exhibits nuclear localization, correlated with strong repression of CLK [18,20]. This indicates that perhaps other kinases (e.g., CKII) phosphorylate CLK, or that PER-independent mechanisms, in particular repression by CWO, are able to compensate for the lack of DBT function [35–37]. CRY has also been shown to act as a repressor of CLK in peripheral clock cells in vivo [72]. Although this repression was shown to require both PER and CRY, the authors conclude that both proteins regulate distinct parts of the cycle (i.e., PER still acts as a repressor after CRY has been degraded by light [13]). Although it is tempting to speculate that PER and CRY form a heterodimer to repress CLK, such a complex has so far only been demonstrated to exist in yeast [68], and PER:CRY interactions in vivo are likely mediated via TIM [69].

In mammals PER proteins (mPER1–3) have been shown to interact with Cryptochromes (mCRY1 and mCRY2), and this interaction is thought to mediate nuclear translocation of both proteins [3,73]. In addition, formation of heterodimers between the different mPER proteins in vitro and within the SCN has been demonstrated [73,74], but their biological function remains elusive. Furthermore, the accompanying report indicates the existence of mPER homodimers (i.e., mPER2:mPER2), and future work will show if they have a similarly important role within the circadian clock, as do the fly PER homodimers described here.

### Conclusions

We provide strong evidence for an important function of the PER:PER homodimer in the *Drosophila* circadian clock. The mutant M560D selectively disrupts PER:PER dimer formation in vivo resulting in a significant reduction of molecular and behavioral rhythmicity. The faulty nuclear localization of M560D proteins in circadian clock neurons strongly support a role for the PER:PER homodimer in nuclear translocation, which is probably closely linked to CKII function. Consistent with these findings, M560D shows a reduced ability to repress *per* transcription in an in vivo transcription assay, resulting in largely abolished *per* transcriptional rhythms.

## Materials and Methods

### Cloning of wild-type and mutant *HA* and *c-myc*-tagged constructs.

The *per* constructs are based on the −1313–34-hs-per, a *pP{CaSpeR-4}* transfection vector with deleted HindIII, PstI, SalI, XhoI, and HpaI restriction sites containing the complete *per* cDNA, a −1313–34 5′-flanking region cloned from the *per* locus, the *hsp70* basal promoter, and a 2.1-kb *per* downstream sequence [48,49]. For addition of the HA- and the *c-myc*-tag, a 4.2-kb XhoI/HindIII fragment (*hsp70* promoter and *per* cDNA) was subcloned into a *pBluescript KS^+^* vector, lacking the BamHI restriction site (further on called pKS-per), followed by addition of an AatII restriction site in front of the tag stop codon, using the QuikChange II XL Site-Directed Mutagenesis Kit (Stratagene) in combination with the oligo nucleotides Aat-S (CCAGACACAGCACGGGGACGTCTAGTAGCCACACCCGC) and Aat-AS (GCGGGTGTGGCTACTAGACGTCCCCGTGCTGTGTCTGG). Annealed and 3′ phosphorylated oligo nucleotides myc-S/myc-AS (TGAGCAGAAGCTGATCAGCGAGGAGGATCTGTACGT, AGAGATCCTCCTCGCTGATCAGCTTCTGCTCAACGT) and HA-S/HA-AS (TTACCCCTACGATGTGCCCGATTACGCCTACGT, AGGCGTAATCGGGCACATCGTAGGGGTAAACGT) were then ligated directly into the AatII restriction site, respectively. After addition of the HindIII/EcoRI fragment of the original −1313–34-hs-per (*per* downstream sequence) to both of the vectors, the BamHI/XbaI fragments, containing the C-terminal part of the *per* cDNA and the 3′ sequences, were exchanged in the −1313–34-hs-per, creating the constructs per-HA and per-c-myc.

Point mutations leading to the exchanges W482E and R345E in the protein sequence were introduced in pKS-per using W482E-S/W482E-AS (AGCTTCGTCAATCCAGAGTCCCGCAAGCTGG, CCAGCTTGCGGGACTCTGGATTGACGAAGCT) and R345E-S/R345E-AS (CCTGGGGCTCACCTTCGAGGAGGCTCCGGAGGAG, CTCCTCCGGAGCCTCCTCGAAGGTGAGCCCCAGG) oligonucleotides, respectively. The XhoI/BamHI fragments (containing the *hsp70* promoter and N-terminal part of the *per* cDNA) of the two mutated pKS-per vectors were then exchanged with the respective wild-type fragment in per-HA and per-c-myc resulting in per-W482E-HA, per-W482E-c-myc, per-R345E-HA, and per-R345E-c-myc. Similarly, the SanDI/BamHI fragment of pAc5.1-V5/His-dPer-M560D [44] was introduced in per-HA and per-c-myc to generate per-M560D-HA and per-M560D-c-myc. To clone the double mutant constructs per-W482E-M560D-HA and per-W482E-M560D-c-myc the wild-type XhoI/SanDI fragment in the two per-M560D vectors was replaced with the same fragment carrying the W482E mutation. All constructs were verified by DNA sequencing.

### Flies and generation of transgenics.

As nontransgenic control flies the wild-type strain *CantonS*, *y Df(1)w* (*y w*), and *w^1118^* flies were used [75]. The original *per^01^* allele [50] was also in a *y w* genetic background to facilitate transgene detection and to increase *luciferase* signals. *P-*element transformation was performed using standard techniques (e.g., [76]) by injecting wild-type and mutant constructs into *y w/y w* (or *y w/Y*); *Ki*Δ*2–3/+* embryos, whereby the Δ*2–3* transposon served as transposase source [77]. G_o_ males were then crossed to *y per^01^ w; Bl/In(2LR)O, Cy* (*CyO*) virgins and the F1 was screened for orange-eyed males. *y per^01^ w; p[w^+^]* males with either *Bl* or *CyO* were then backcrossed to *y per^01^ w;Bl/CyO* virgins. If the transgene did not map to Chromosome *2, y per^01^ w; p[w^+^]* males were subsequently crossed to a *y per^01^ w* strain containing a dominant marker and balancer chromosome for Chromosome *3*. At least five independent transgenic lines were established for each construct, and results were confirmed with at least three independent lines. For *per-M560D-c-myc* we originally isolated only one homozygous lethal line mapping to Chromosome *3* (10-2-1) and one homozygous viable line mapping to Chromosome *4* (10-2-2) (see Table S1). We therefore mobilized the *P-*element in line 10-2-2 by backcrossing to the homozygous *Ki*Δ*2–3* strain and isolating homozygous viable inserts mapping to Chromosome *2* using standard crossings [77]. One of these lines (*per-M560D-c-myc*:10-2-2J5) was used to create a double-homozygous stock with *per-M560D-HA*:9–8 for CoIP experiments using standard crosses and balancer chromosomes. To create the double-homozygous flies expressing wild-type *per-c-myc* and *per-HA* constructs lines 1-5-2 and 2-2-2 were used.

### Behavior analysis.

Two- to 3-d-old individual adult male flies were loaded in small glass tube sealed at one end with food (5% sucrose, 2% agar) and closed at the other end by cotton. The locomotor activity is detected by an automated infrared beam monitoring system (Trikinetics) for 4–7 d in a 12-h:12-h LD cycle and then in DD for another 7 d. Daily average histograms and actograms were plotted using the fly toolbox and MATLab software [78]. The free-running period was calculated using the Autocorrelation function. In this study all flies with an Rhythmicity Statistics (RS) value >1 were considered as rhythmic (see [78,79] for how this cut-off was determined).

### Immunohistochemistry.

Anti-PER antibody stainings were performed as previously described [80]. Prior to collection at ZT16, ZT20 and ZT2, the flies from different strains were entrained for at least 2 d under 12-h:12-h LD conditions. Whole-mounted brains were dissected and collected in Ringer solution before being fixed in 4% paraformaldehyde at 4 °C overnight. After fixation, the samples were washed ten times with 0.1 M phosphate buffer (pH 7.4) and three times in PBS with 1% Triton X-100 (PBS-T) at room temperature (RT). The brains were then blocked with 10% goat serum in PBS-T for 2 h in RT and stained with pre-absorbed polyclonal rabbit anti-PER in PBS-T at 1:1,000 dilution. After washing three times by PBS-T, the samples were incubated at 4 °C overnight with goat-anti-rabbit antibody conjugated with fluorophore, AlexaFluor 488 nm (Molecular Probes) diluted 1:300 in PBS-T. For double-labeling, samples were washed with PBS-T and incubated with blocking serum and polyclonal rabbit anti-PDF antibody diluted 1:1,000 in PBS-T at 4 °C overnight. The samples were then treated with goat-anti-rabbit antibody conjugated with fluorophore, AlexaFluor 594 nm (Molecular Probes) diluted 1:300 in PBS-T at 4 °C overnight. Brains were washed three times in PBS-T and water before being mounted in Vectashield. Samples were stored at 4 °C until examination under a LSM-510 META confocal microscope (Zeiss).

PDF signals in the LNvs were used as cytoplasmic marker. Yellow or orange staining of outside the nucleus caused by co-expression of PDF and PER (green) was scored as “cytoplasmic PER” (C). Green signals in the centre of LNvs were scored as “nuclear PER” (N), and neurons with yellow in periphery and green in the centre as “nuclear and cytoplasmic” (N/C). See legends of Figures S3 and S4 for further details.

### CoIPs.

CoIPs were performed as described [69]. Briefly, adult flies from the HA- and *c-myc*-tagged strains were entrained to a 12-h:12-h LD cycle for 2 d. At ZT16, ZT20 and ZT2, 6 ml of flies were collected in liquid nitrogen and frozen in −80 °C until homogenization. Fly heads were separated by repeated vortexing/cooling in liquid nitrogen. 400 μl of fly heads were then isolated using a 0.45-mm/0.14-mm prechilled metal mesh. Heads were then homogenized in 400-μl of Extraction Buffer (20 mM Hepes [pH 7.5], 100 mM KCl, 1 mM dithiothreitol, 5% glycerol, 0.05% Nonidet P40, 1× Complete Protease Inhibitor [Roche]). 20 μl of head extract were boiled with 5 μl of 5× SDS loading buffer as input control. Protein G Sepharose fast flow beads (Amersham) were coated with anti-MYC antibody (5 μl anti-MYC antibody [Covance Inc.] + 20 μl beads/sample in 1 ml extraction buffer, 4 °C for 1 h) and incubated with the head extracts for 16 h at 4 °C. Beads were spun down by centrifugation, and 20 μl of supernatant were boiled with 5 μl of 5× SDS loading buffer as supernatant control. The pulled-down beads were washed three times with 750 μl Extraction Buffer before being resuspended in 30 μl 1× SDS loading buffer for western blot.

### Western blotting.

Flies of the indicated genotypes were first kept in LD cycles for at least 3 d and collected on dry ice during the indicated ZT in LD. Preparations of head extracts and protein blots were performed as described [81]. Twenty-five fly heads for each genotype/time point were collected and homogenized with 40 μl of Extraction buffer (20 mM HEPES [pH 7.5], 100 mM KCl, 5% glycerin, 10 mM EDTA, 0.1% Triton-X 100, 20 mM β-glycerophosphat, 0.1 mM Na3VO4, 1× Complete Protease Inhibitor [Roche]) and centrifuged at 4 °C at 13,000 rpm. The supernatants were transferred and boiled with 1 × SDS loading buffer for 5 min before loading and running on 4.5%/6.0% SDS-PAGE overnight at 55–70 V. CoIP samples were separated on the same gels, and proteins were blotted to Nitrocellulose at 500 mA for 1 h with a Semi-dry electro blotting unit (Fisherbrand) according to the manual. After blotting, the nitrocellulose membranes were blocked with 5% nonfat milk in TBS-T at room temperature for 1 h. First antibodies (5% nonfat milk/TBS-T) were diluted and incubated at 4 °C overnight, followed by secondary antibody (5% nonfat milk/TBS-T) incubation at room temperature for 2 h. Dilution of first antibodies were 1:10,000 for rabbit anti-PER, 1:2,000 for rat anti-TIM, and 1:1,000 for mouse anti HA-tag (MMS-101p, Covance). The dilutions of HRP-conjugated secondary antibodies were 1:100,000 for goat-anti-rabbit IgG, 1:25,000 for goat anti rat IgG, and 1:5,000 for goat-anti-mouse IgG. ECL substrates were used, and X-ray films were developed according to manufacturer's instructions.

### Luciferase monitoring.

Bioluminescence assays were performed as previously described [57]. Briefly, 2–3-d-old individual male flies carrying the *plo per-luciferase* transgene were ether-anesthetized and loaded in a 96-well micro titer plate in which each of the well contains 100 μl of 5% sucrose, 1% agar, and 15 mM luciferin. Flies were measured in a Packard Topcount Multiplate Scintillation Counter at 25 °C and 12-h:12-h LD cycles for 7 d. Data were plotted using BRASS (biological rhythms analysis software system) Version 2.1.3. [82]. Period values and rhythm significance (rel-amp error) were calculated using FFT-NLLS analysis as described in [83]. In the current analysis flies with relative-amplitude errors (rel-amp) <0.7 and period values of 24 ± 2 h were considered as rhythmic. Rel-amp errors <0.7 indicate that the rhythms in the bioluminescence data determined by the FFT-NLLS analysis are due to rhythmic gene expression with 95% confidence [57].

## Supporting Information

Figure S1 mRNA Expression of *period* Trangenics in Fly Heads vRelative abundance of c-myc tagged per mRNA in fly heads of different transgenic fly strains at ZT15. RNA was extracted from 30 fly heads per fly strain using Trizol reagent (Peqlab) according to manufacturer's instructions. RNA was converted to cDNA using the QuantiTect Reverse Transcription Kit (Qiagen). The QuantiTect SYBR Green PCR kit (Qiagen) was then used for the quantitative PCR reaction in a LightCycler (Roche). For the c-myc containing *per* cDNA two *per* specific oligonucleotides, *per1* ACCGAAAGCTGAAGAGCATG and *per2* GACCCCAAGCACCGAAAGCTG were used each in combination with the c-myc specific oligonucleotide c-myc1, CCTCGCTGATCAGCTTCTGCT, to discriminate transgene encoded RNA from the *X*-chromosomal derived (untagged) *per* mRNA. Resultant cDNA quantities were normalized to α*-Tubulin84B* measured in each respective sample (oligonucleotides tub1 TCCTTGTCGCGTGTGAAACA and tub2 GTGCTTGCCAGCTCCAGTCT). Two independent cDNA samples were prepared for each fly strain and each sample was analyzed four times using *per1* and *per2* twice each, in combination with c-myc1. Average abundance in each fly line was normalized to the highest average value (which was set to 1), and the standard error of the mean was calculated.
(139 KB PDF)

Figure S2 Quantification of PER Protein Expression and Migration Behavior The average of those experiments performed as shown in Figure 2 is plotted. Genotypes: *per-wt, 2-2-2, 1-5-2; per-M560D, 9-4-2, 10-2-2:J5*.
(A) Band intensities (mean gray value) of each lane were determined by ImageJ. The ratio to the mean value were obtained by dividing individual intensity to the daily average value of each genotype. The amplitude of each line: *per-wt*: 1.5-fold and *per-M560D*: 1.2-fold.
(B) In order to determine the relative migration distance of PER protein, the most concentrated point of a band, center of mass, in the *y* dimension of each lane (in pixels) was determined by the ImageJ system. Midpoint, the average value of all lanes was calculated and used as migrating reference point. The distance to midpoint was then determined by subtracting each individual center of mass value from midpoint. More positive distance to midpoint values indicate the faster migrating species and vice versa. Error bars indicate standard error of the mean.
(150 KB PDF)

Figure S3 PER Positive Clock Neurons Are Reduced in the M560D Mutant Quantification of confocal images shown in Figure 6. The percentages were calculated by dividing the number of PER positive neurons by that of PDF positive neurons for individual hemispheres of each genotype. The means were then calculated and plotted for each genotype and ZT time. Student's *t*-test was performed to determine significance of the reduction of PER positive neurons (indicated by an asterisk [*], *p* < 0.05). For number of brains analyzed see Figure 6.
(108 KB PDF)

Figure S4 Quantification of PER Subcellular Localization Confocal images as shown in Figure 6 were quantified as follows: Percentages were calculated by dividing the number of PER positive neurons showing staining in nucleus (N), in both nucleus and cytoplasm (N/C), or cytoplasm (C), by that of PDF positive neurons for the individual hemispheres of each genotype. Mean and standard error of the mean was then calculated and plotted for each genotype and ZT. One-way ANOVA and the Bonfferoni post test were performed to compare the difference among all groups at a given time point. Identical alphabetic symbols were assigned to individual groups based on statistical significance (*p* > 0.05). Groups with the same symbol are not significantly different. For number of brains analyzed see Figure 6.
(152 KB PDF)

Table S1 Locomoter Activity Rhythms of Controls and In Vitro Mutagenized PER Mutants Locomotor rhythms of individual nontransgenic and transgenic flies were recorded as described in Materials and Methods. Flies were entrained to 12-h:12-h LD cycles at the indicated temperature for at least 4 d before being released to constant darkness at the same temperature. Transgenic flies were homozygous for the respective *P-*element, except when the insertion caused lethality when homozygous (indicated by the CyO or TM6B balancer symbol in the “line” column) where only one copy of the transgene was present. Free-running period values (Period) and rhythm strength (RS) were calculated by Autocorrelation (see Materials and Methods for details).
(284 KB DOC)

Table S2 Rescue of *per^01^* Bioluminescence Rhythms by Wild-Type and M560D Mutant PER Proteins Bioluminescence rhythms of *per^01^* flies carrying the *plo* reporter gene and either wild-type PER or M560D PER encoding constructs were recorded as described in Materials and Methods. Flies were kept in 12-h:12-h LD cycles at 25 °C for the entire length of the experiment (5 d). Period values and rhythmicity was determined by performing an FFT-NLLS analysis with the raw data. Flies with rel-amp errors <0.7 and period values of 24 ± 2 h were considered as rhythmic (Materials and Methods).
(38 KB DOC)

## Abbreviations

CLK: Clock
CoIP: co-immunoprecipitation
CWO: Clockwork Orange
CYC: Cycle
*dbt,*: *double-time;*
DD: constant darkness
LD: light–dark
LN: lateral clock neuron
PER: Period
TIM: Timeless
