# Supplementary material for: A Role for the PERIOD:PERIOD Homodimer in the Drosophila Circadian Clock
Source: PLoS Biol. 2009 Apr 28;7(4):e1000003. doi: 10.1371/journal.pbio.1000003 (PMC2671555; doi:10.1371/journal.pbio.1000003)
Supplement: Supplementary file 1 [file pbio.1000003.sg001.pdf]

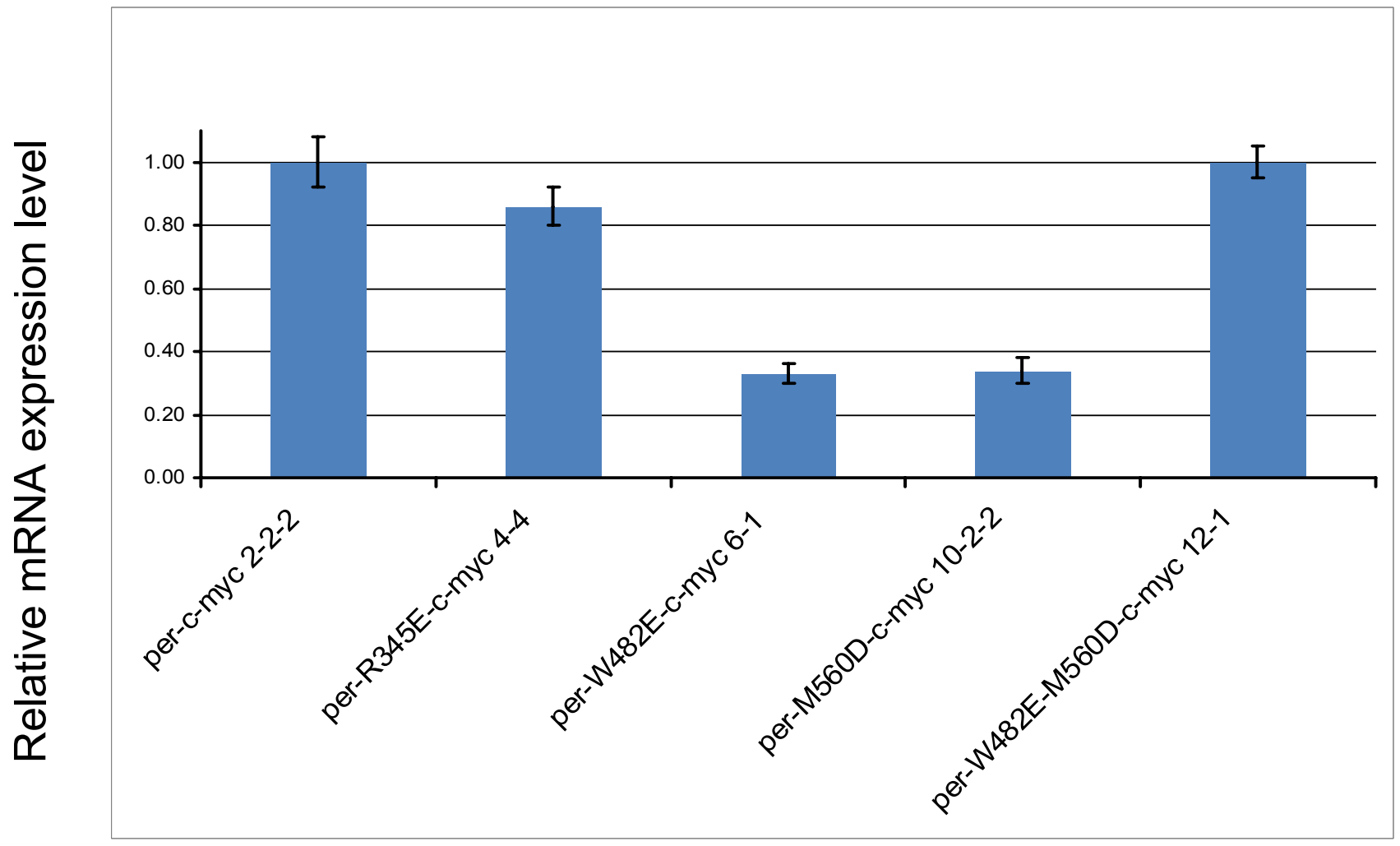


**Figure S1: mRNA expression of *period* trangenics in fly heads:**

Relative abundance of c-myc tagged per mRNA in fly heads of different transgenic fly strains at ZT15. RNA was extracted from 30 fly heads per fly strain using Trizol reagent (Peqlab, Erlangen, Germany) according to manufacturer's instructions. RNA was converted to cDNA using the QuantiTect Reverse Transcription Kit (Qiagen, Hilden, Germany). The QuantiTect SYBR Green PCR Kit (Qiagen) was then used for the quantitative PCR reaction in a LightCycler (Roche, Mannheim, Germany). For the c-myc containing *per* cDNA two *per* specific oligonucleotides, *per1* ACCGAAAGCTGAAGAGCATG and *per2* GACCCCAAGCACCGAAAGCTG were used each in combination with the c-myc specific oligonucleotide c-myc1, CCTCGCTGATCAGCTTCTGCT, to discriminate trangene encoded RNA from the *X*-chromosomal derived (untagged) *per* mRNA. Resultant cDNA quantities were normalized to *α-Tubulin84B* measured in each respective sample (oligonucleotides tub1 TCCTTGTCGCGTGTGAAACA and tub2 GTGCTTGCCAGCTCCAGTCT). Two independent cDNA samples were prepared for each fly strain and each sample was analyzed four times using *per1* and *per2* twice each, in combination with c-myc1. Average abundance in each fly line was normalized to the highest average value (which was set to 1) and the SEM was calculated.
