# Supplementary material for: A Role for the PERIOD:PERIOD Homodimer in the Drosophila Circadian Clock
Source: PLoS Biol. 2009 Apr 28;7(4):e1000003. doi: 10.1371/journal.pbio.1000003 (PMC2671555; doi:10.1371/journal.pbio.1000003)
Supplement: Supplementary file 2 [file pbio.1000003.sg002.pdf]

A

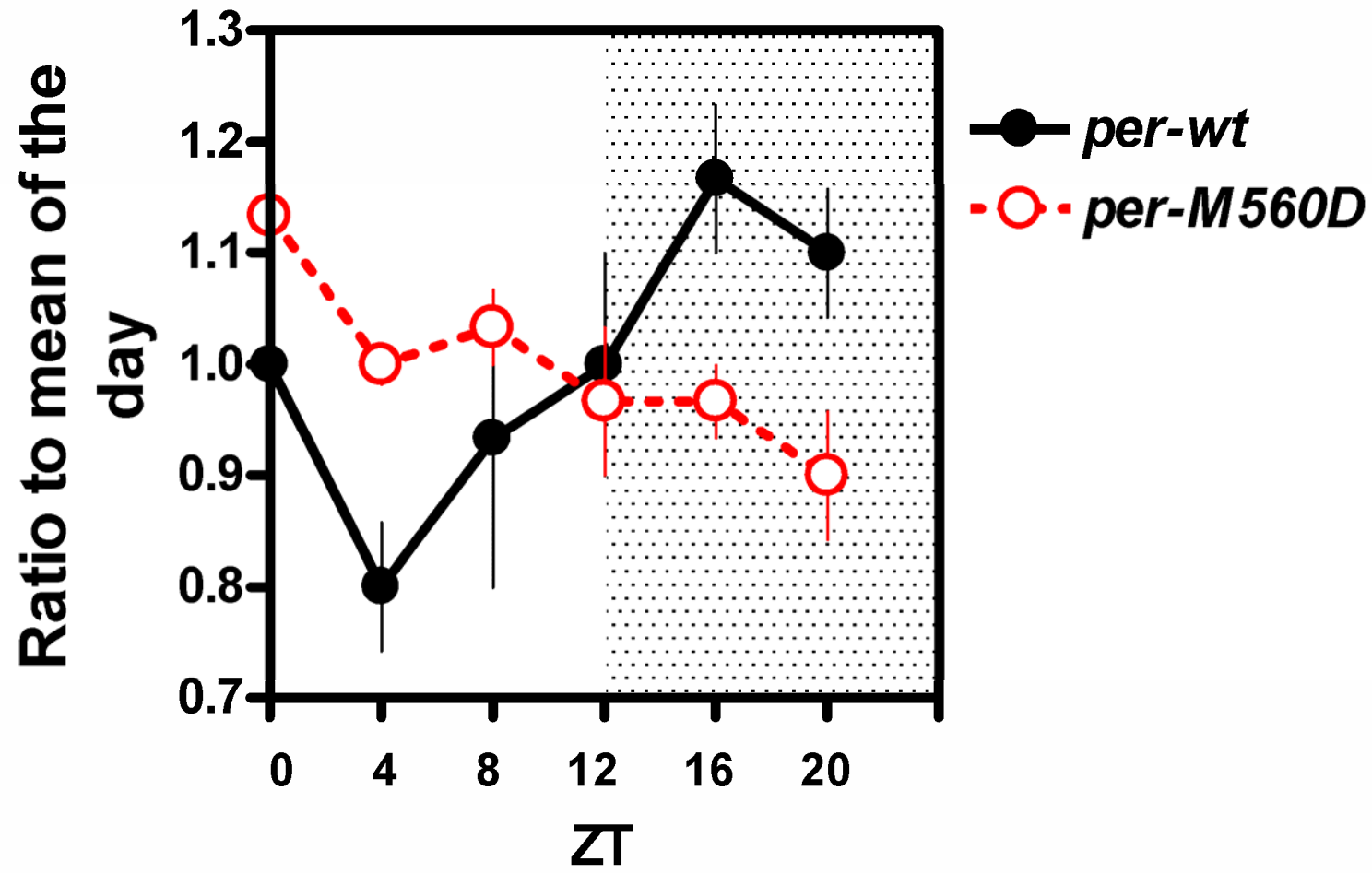

B

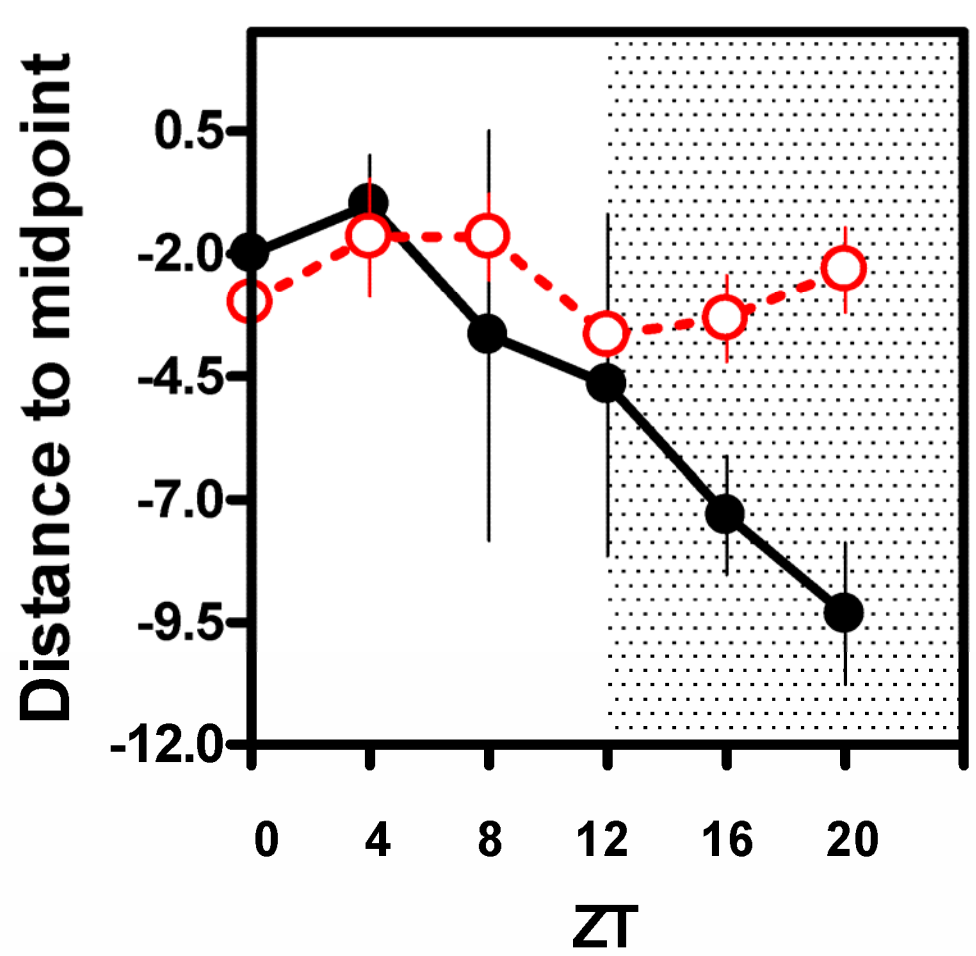

Figure S2. Quantification of PER protein expression and migration behavior.
The average of those experiments performed as shown in Figure 2is plotted. Genotypes: *per-wt: 2-2-2, 1-5-2, per-M560D: 9-4-2, 10-2-2:J5*. **A.** Band intensities (mean gray value) of each lane were determined by ImageJ. The ratio to the mean value were obtained by dividing individual intensity to the daily average value of each genotype. The amplitude of each line: *per-wt*:1.5 fold and *per-M560D*:1.2 fold. **B.** In order to determine the relative migration distance of PER protein, the most concentrated point of a band, **centre of mass**, in the Y-dimension of each lane (in pixels) was determined by the ImageJ system. **Midpoint**, the average value of all lanes was calculated and used as migrating reference point. The **distance to midpoint** was then determined by subtracting each individual center of mass value from midpoint. More positive distance to midpoint values indicate the faster migrating species and *vice versa*. Error bars indicates SEM.
