# Supplementary material for: A Role for the PERIOD:PERIOD Homodimer in the Drosophila Circadian Clock
Source: PLoS Biol. 2009 Apr 28;7(4):e1000003. doi: 10.1371/journal.pbio.1000003 (PMC2671555; doi:10.1371/journal.pbio.1000003)
Supplement: Supplementary file 3 [file pbio.1000003.sg003.pdf]

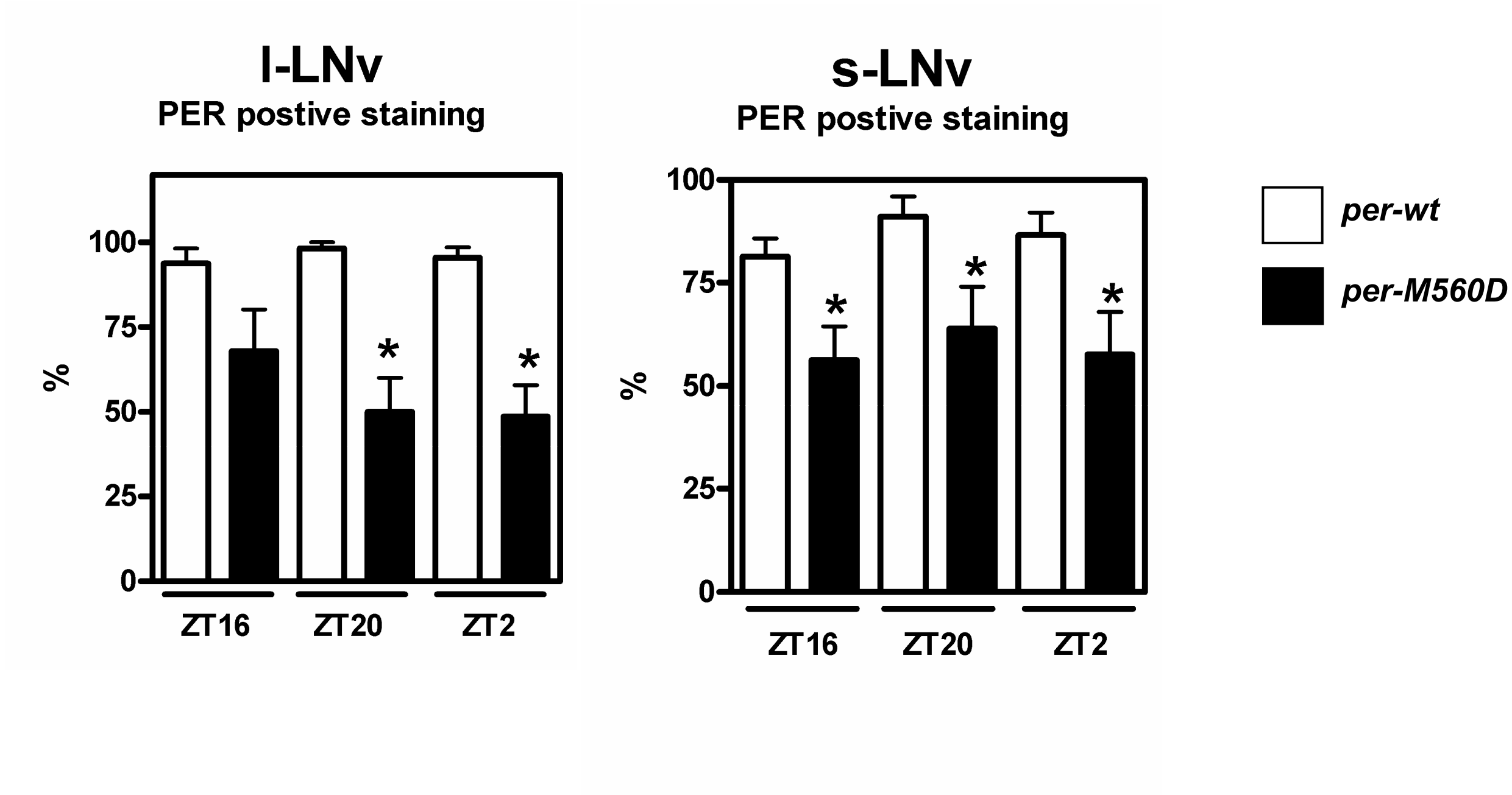

Figure S3. PER positive clock neurons are reduced in the M560D mutant.
Quantification of confocal images shown in Figure 6. The percentages were calculated by dividing the number of PER positive neurons by that of PDF positive neurons for individual hemispheres of each genotype. The means were then calculated and plotted for each genotype and ZT time. *Student's t-test* was performed to determine significance of the reduction of PER positive neurons (indicated by an asterisk (*), $p<0.05$). For number of brains analyzed see Figure 6.
