# Supplementary material for: A Role for the PERIOD:PERIOD Homodimer in the Drosophila Circadian Clock
Source: PLoS Biol. 2009 Apr 28;7(4):e1000003. doi: 10.1371/journal.pbio.1000003 (PMC2671555; doi:10.1371/journal.pbio.1000003)
Supplement: Supplementary file 4 [file pbio.1000003.sg004.pdf]

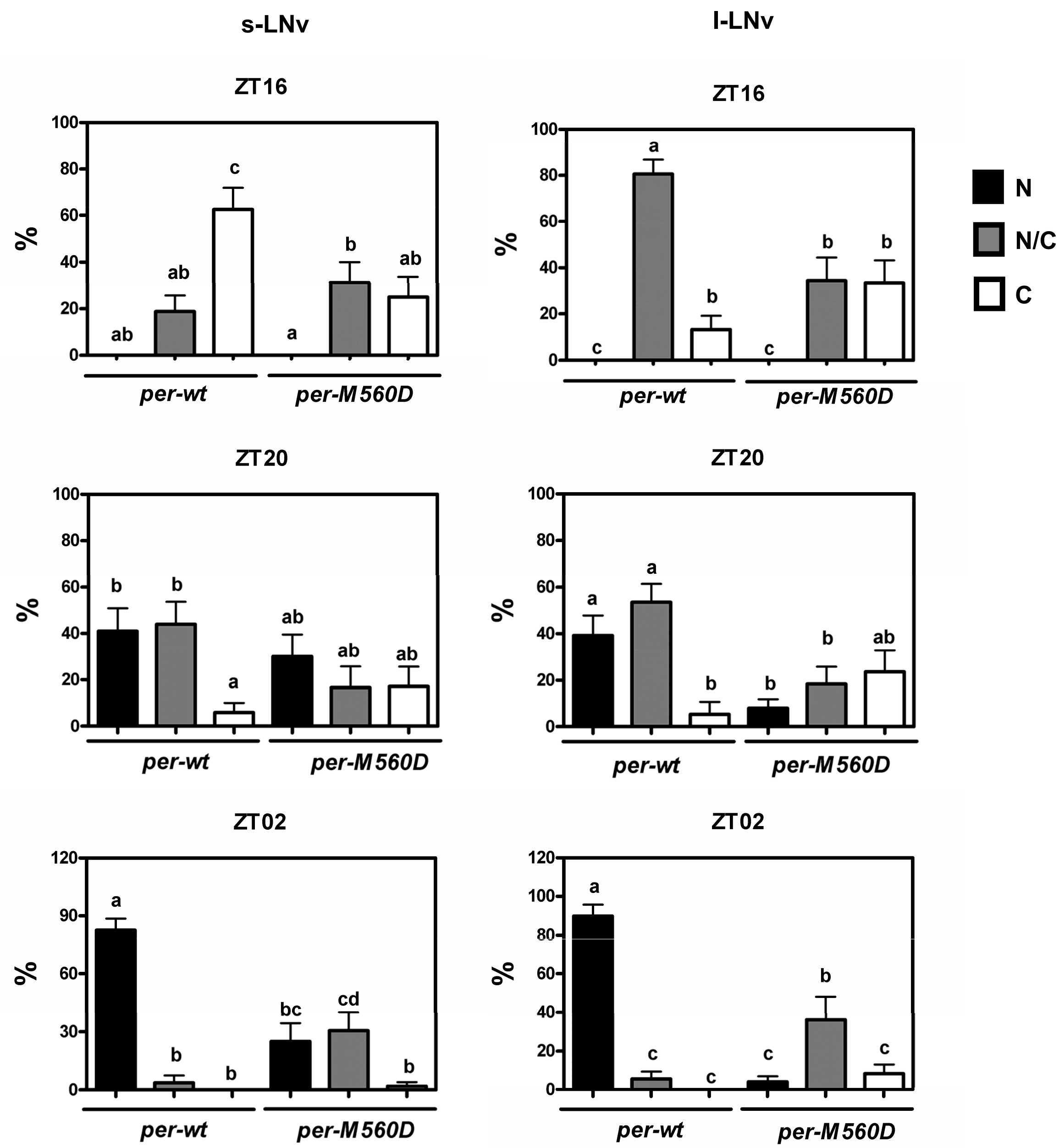

Figure S4.Quantification of PER subcellular localization.
Confocal images as shown in Figure 6 were quantified as follows: Percentages were calculated by dividing the number of PER positive neurons showing staining in nucleus (N), in both nucleus and cytoplasm (N/C) or cytoplasm (C), by that of PDF positive neurons for the individual hemispheres of each genotype. Mean and SEM was then calculated and plotted for each genotype and ZT. *One-way ANOVA* and the Bonfferoni post-test were performed to compare the difference among all groups at a given time point. Identical alphabetic symbols were assigned to individual groups based on statistical significance (p>0.05). Groups with the same symbol are not significantly different. For number of brains analyzed see Figure 6.
