# Supplementary material for: A Role for the PERIOD:PERIOD Homodimer in the Drosophila Circadian Clock
Source: PLoS Biol. 2009 Apr 28;7(4):e1000003. doi: 10.1371/journal.pbio.1000003 (PMC2671555; doi:10.1371/journal.pbio.1000003)
Supplement: Supplementary file 5 [file pbio.1000003.st001.doc]

**Table S1: Locomoter activity rhythms of controls and *in vitro* mutagenized PER mutants**

| **18°C** |  |  |  |  |  |  |  |
| --- | --- | --- | --- | --- | --- | --- | --- |
| **Genotype** | **line** | **rhythmic** | **Period (hr)** | **SEM** | **RS** | **SEM** | **n** |
| *CantonS* |  | 96.2% | 23.6 | 0.4 | 3.3 | 0.2 | 26 |
| *y w* |  | 71.4% | 23.7 | 0.2 | 2.5 | 0.3 | 28 |
| *per-HA* | 1-5-2 | 68.8% | 22.1 | 0.3 | 2.3 | 0.2 | 16 |
|  | 1-8 | 81.3% | 21.7 | 0.4 | 2.2 | 0.1 | 16 |
| *per-c-myc* | 2-2-2 | 100.0% | 23.2 | 0.3 | 2.7 | 0.3 | 16 |
|  | 2-6 | 80.0% | 23.0 | 0.2 | 2.6 | 0.3 | 15 |
| *R345E-HA* | 3-1 | 50.0% | 22.9 | 0.9 | 1.6 | 0.3 | 16 |
|  | 3-4 | 0.0% |  |  |  |  | 16 |
| *R345E-c-myc* | 4-4 | 13.3% | 24.1 | 2.3 | 2.7 | 0.6 | 15 |
|  | 4-9-2 | 31.3% | 24.2 | 0.9 | 1.7 | 0.2 | 16 |
| *W482E-HA* | 5-2/CyO | 18.8% | 23.4 | 3.1 | 1.8 | 0.3 | 16 |
|  | 5-3 | 18.8% | 26.3 | 0.3 | 1.7 | 0.6 | 16 |
| *W482E-c-myc* | 6-1 | 18.8% | 22.2 | 1.5 | 2.2 | 0.4 | 16 |
|  | 6-3-2 | 18.8% | 24.4 | 2.2 | 2.2 | 0.4 | 16 |
| *M560D-HA* | 9-2 | 12.5% | 25.7 | 1.4 | 1.9 | 0.9 | 16 |
|  | 9-4-2 | 50.0% | 21.7 | 0.5 | 2.0 | 0.1 | 16 |
| *M560D-c-myc* | 10-2-1/TM6B | 25.0% | 23.3 | 1.6 | 1.5 | 0.7 | 16 |
|  | 10-2-2 | 37.5% | 25.1 | 0.5 | 1.3 | 0.3 | 16 |
| *W482E-M560D-HA* | 11-6 | 12.5% | 23.3 | 0.3 | 2.4 | 0.7 | 16 |
|  | 11-7 | 13.3% | 25.4 | 1.9 | 1.5 | 0.1 | 16 |
| *W482E-M560D-myc* | 12-1 | 37.5% | 26.3 | 0.8 | 1.8 | 0.4 | 16 |
|  | 12-2 | 37.5% | 24.5 | 1.7 | 1.7 | 0.4 | 16 |
|  |  |  |  |  |  |  |  |
| **25°C** |  |  |  |  |  |  |  |
| **Genotype** | **line** | **rhythmic** | **Period (hr)** | **SEM** | **RS** | **SEM** | **n** |
| *y per01 w* |  | 0% |  |  |  |  | 24 |
| *y w* |  | 87.0% | 23.4 | 0.2 | 2.9 | 0.3 | 23 |
| *per-HA* | 1-3/TM6B | 69.2% | 23.1 | 0.3 | 3.2 | 0.5 | 13 |
|  | 1-5-1 | 66.7% | 25.2 | 1.4 | 2.2 | 1.1 | 3 |
|  | 1-5-2 | 85.7% | 23.8 | 0.4 | 2.8 | 0.5 | 7 |
|  | 1-6/TM6B | 87.5% | 22.9 | 0.2 | 3.2 | 0.1 | 16 |
|  | 1-7/CyO | 87.5% | 22.6 | 0.1 | 3.6 | 0.6 | 8 |
|  | 1-8 | 75.0% | 22.6 | 0.5 | 2.0 | 0.3 | 8 |
|  | 1-9 | 57.1% | 22.9 | 0.2 | 2.6 | 0.3 | 15 |
|  | 1-10/CyO | 37.5% | 25.8 | 1.0 | 2.0 | 0.5 | 8 |
|  | 1-11 | 50.0% | 22.5 | 0.3 | 2.3 | 0.3 | 8 |
| *per-c-myc* | 2-2-1 | 75.0% | 23.0 | 0.5 | 2.4 | 0.2 | 8 |
|  | 2-2-2 | 75.0% | 23.2 | 0.4 | 1.8 | 0.2 | 8 |
|  | 2-3 | 50.0% | 24.0 | 0.8 | 2.1 | 0.3 | 8 |
|  | 2-4-1 | 50.0% | 23.6 | 1.4 | 2.0 | 0.2 | 8 |
|  | 2-4-2 | 60.0% | 22.6 | 0.2 | 2.2 | 0.5 | 5 |
|  | 2-5 | 66.7% | 22.8 | 0.1 | 2.2 | 0.3 | 6 |
|  | 2-6 | 100.0% | 24.0 | 0.3 | 2.7 | 0.3 | 13 |
|  | 2-7/CyO | 62.5% | 22.5 | 0.3 | 2.9 | 0.4 | 8 |
| *per-c-myc; per-HA* | 1-5-2;2-2-2 | 84.2% | 23.2 | 0.5 | 2.7 | 0.2 | 38 |
|  |  |  |  |  |  |  |  |
|  |  |  |  |  |  |  |  |
| *per-R345E-HA* | 3-1 | 37.5% | 24.2 | 2.0 | 2.0 | 0.1 | 8 |
|  | 3-2 | 12.5% | 26.0 |  | 0.8 |  | 8 |
|  | 3-3/TM6B | 37.5% | 25.8 | 2.2 | 1.3 | 0.3 | 8 |
|  | 3-4 | 28.6% | 23.0 | 0.0 | 1.6 | 0.4 | 8 |
|  | 3-5 | 0.0% |  |  |  |  | 8 |
|  | 3-6 | 0.0% |  |  |  |  | 8 |
|  | 3-7/CyO | 16.7% | 22.3 |  | 1.5 |  | 1 |
|  | 3-8 | 0.0% |  |  |  |  | 8 |
|  | 3-10/CyO | 37.5% | 24.5 | 1.7 | 2.2 | 0.2 | 8 |
| *per-R345E-c-myc* | 4-4 | 12.5% | 22.5 |  | 2.0 |  | 8 |
|  | 4-5-1 | 0.0% |  |  |  |  | 7 |
|  | 4-6 | 12.5% | 23.8 |  | 2.5 |  | 8 |
|  | 4-9-1 | 25.0% | 25.3 | 2.3 | 1.3 | 0.0 | 8 |
|  | 4-9-2 | 37.5% | 21.4 | 0.7 | 2.1 | 0.4 | 8 |
| *per-W482E-HA* | 5-2/CyO | 0.0% |  |  |  |  | 7 |
|  | 5-3 | 12.5% | 26.8 |  | 1.8 |  | 8 |
| *per-W482E-c-myc* | 6-1 | 25.0% | 27.5 | 0.3 | 1.5 | 0.1 | 8 |
|  | 6-2 | 0.0% |  |  |  |  | 8 |
|  | 6-3-1 | 0.0% |  |  |  |  | 4 |
|  | 6-4 | 0.0% |  |  |  |  | 8 |
|  | 6-5/CyO | 0.0% |  |  |  |  | 16 |
|  | 6-6 | 9.1% | 26.8 |  | 2.4 |  | 11 |
| *per-M560D-HA* | 9-1 | 12.5% | 24.0 |  | 2.0 |  | 8 |
|  | 9-2 | 25.0% | 22.6 | 0.8 | 4.7 | 1.3 | 8 |
|  | 9-4-1 | 37.5% | 23.1 | 1.8 | 2.0 | 0.3 | 8 |
|  | 9-4-2 | 50.0% | 23.8 | 0.4 | 2.6 | 0.3 | 8 |
|  | 9-5/TM6B | 42.9% | 23.3 | 1.0 | 1.3 | 0.3 | 7 |
|  | 9-6 | 0.0% |  |  |  |  | 6 |
|  | 9-8 | 37.5% | 23.9 | 1.6 | 1.7 | 0.3 | 8 |
| *per-M560D-c-myc* | 10-2-1/TM6B | 62.5% | 25.4 | 0.4 | 2.5 | 0.3 | 16 |
|  | 10-2-2 | 50.0% | 24.6 | 0.7 | 2.4 | 0.4 | 16 |
|  | 10-2-2:J5 | 66.7% | 24.8 | 0.3 | 2.4. | 0.2 | 12 |
| *per-M560D-c-myc; per-M560D-HA* | 10-2-2:J5;9-8 | 62.5% | 22.3 | 1.0 | 2.2 | 0.2 | 32 |
|  | 10-2-2:J3;9-4-2 | 45.5% | 23.4 | 3.1 | 2.0 | 0.1 | 11 |
|  |  |  |  |  |  |  |  |
|  |  |  |  |  |  |  |  |
| *per-W482D-M560D-HA* | 11-3 | 0.0% |  |  |  |  | 7 |
|  | 11-4-1/CyO | 22.2% | 25.2 | 1.9 | 1.6 | 0.4 | 9 |
|  | 11-4-3/CyO | 0.0% |  |  |  |  | 8 |
|  | 11-5 | 0.0% |  |  |  |  | 8 |
|  | 11-6 | 0.0% |  |  |  |  | 8 |
|  | 11-7 | 12.5% | 27.5 |  | 2.2 |  | 8 |
|  | 11-8 | 0.0% |  |  |  |  | 8 |
|  | 11-10 | 0.0% |  |  |  |  | 8 |
| *per-W482D-M560D-c-myc* | 12-1 | 12.5% | 25.8 |  | 2.3 |  | 8 |
|  | 12-2 | 0.0% |  |  |  |  | 8 |
|  | 12-4 | 0.0% |  |  |  |  | 8 |
|  | 12-6/TM6B | 0.0% |  |  |  |  | 8 |
|  | 12-7 | 0.0% |  |  |  |  | 8 |
|  | 12-8/CyO | 25.0% | 22.9 | 1.6 | 2.7 | 0.3 | 8 |
|  |  |  |  |  |  |  |  |
| **29°C** |  |  |  |  |  |  |  |
| **Genotype** | **line** | **rhythmic** | **Period (hr)** | **SEM** | **RS** | **SEM** | **n** |
| *Canton* |  | 100% | 23.6 | 0.2 | 3.3 | 0.4 | 6 |
| *w^1118^* |  | 100% | 23.6 | 0.1 | 3.1 | 0.6 | 7 |
| *y w* |  | 93.6 | 23.8 | 0.1 | 5.5 | 0.4 | 16 |
| *per-HA* | 1-5-2 | 93.8% | 22.3 | 0.2 | 3.3 | 0.3 | 16 |
|  | 1-8 | 87.5% | 21.9 | 0.1 | 3.4 | 0.5 | 16 |
| *per-c-myc* | 2-2-2 | 81.3% | 22.3 | 0.1 | 3.6 | 0.4 | 16 |
|  | 2-6 | 87.5% | 22.9 | 0.2 | 3.9 | 0.4 | 16 |
| *per-R345E-HA* | 3-1 | 6.3% | 26.5 |  | 2.3 |  | 16 |
|  | 3-2 | 0.0% |  |  |  |  | 8 |
|  | 3-4 | 0.0% |  |  |  |  | 8 |
| *per-R345E-c-myc* | 4-4 | 0.0% |  |  |  |  | 16 |
|  | 4-9-2 | 0.0% |  |  |  |  | 16 |
| *per-W482E-HA* | 5-2/CyO | 0.0% |  |  |  |  | 16 |
|  | 5-3 | 0.0% |  |  |  |  | 16 |
| *per-W482E-c-myc* | 6-1 | 0.0% |  |  |  |  | 16 |
|  | 6-3-2 | 0.0% |  |  |  |  | 15 |
|  | 6-5/CyO |  |  |  |  |  | 0 |
| *per-M560D-HA* | 9-2 | 43.8% | 23.3 | 0.5 | 1.9 | 0.4 | 16 |
|  | 9-4-2 | 62.5% | 21.9 | 0.1 | 3.2 | 0.5 | 16 |
| *per-M560D-c-myc* | 10-2-1/TM6B | 68.8% | 24.9 | 0.2 | 3.1 | 0.5 | 16 |
|  | 10-2-2 | 56.3% | 24.3 | 0.6 | 1.9 | 0.2 | 16 |
| *per-W482E-M560D-HA* | 11-6 | 0.0% |  |  |  |  | 16 |
|  | 11-7 | 16.7% | 27.5 | 2.5 | 1.3 | 0.3 | 12 |
| *per-W482E-M560D-c-myc* | 12-1 | 6.3% | 26.3 |  | 2.1 |  | 16 |
|  | 12-2 | 6.3% | 27.0 |  | 1.2 |  | 16 |

Locomotor rhythms of individual non-transgenic and transgenic flies were recorded as described in Materials and Methods. Flies were entrained to 12 hr: 12 LD cycles at the indicated temperature for at least 4 days before being released to constant darkness at the same temperature. Transgenic flies were homozygous for the respective *P-*element, except when the insertion caused lethality when homozygous (indicated by the CyO or TM6B balancer symbol in the ‘line’ column) where only one copy of the transgene was present. Free-running period values (Period) and rhythm strength (RS) were calculated by Autocorrelation (see Materials and Methods for details)
