# Supplementary material for: A Role for the PERIOD:PERIOD Homodimer in the Drosophila Circadian Clock
Source: PLoS Biol. 2009 Apr 28;7(4):e1000003. doi: 10.1371/journal.pbio.1000003 (PMC2671555; doi:10.1371/journal.pbio.1000003)
Supplement: Supplementary file 6 [file pbio.1000003.st002.doc]

**Table S2: Rescue of *per^01^*** **bioluminescence rhythms by wild-type and M560D mutant PER proteins**

| Genotype | Line | n | Period (hr) | % rhythmic | rel-amp |
| --- | --- | --- | --- | --- | --- |
| *per^01^* wild-type *per* |  |  |  |  |  |
|  | 1-5-2 | 12 | 24.4 ± 0.2 | 75 | 0.45 ± 0.04 |
|  | 1-8 | 12 | 24.3 ± 0.3 | 75 | 0.48 ± 0.03 |
|  | 2-2-2 | 12 | 24.3 ± 0.1 | 100 | 0.36 ± 0.03 |
|  | 2-6 | 12 | 24.4 ± 0.1 | 83 | 0.49 ± 0.04 |
|  | **all** | **48** | **24.4 ± 0.1** | **83** | **0.44 ± 0.02** |
| *per^01^* *M560D* *per* |  |  |  |  |  |
|  | J5 | 12 | 24.2 ± 0.2 | 33 | 0.61 ± 0.06 |
|  | J6 | 12 | 24.2 ± 0.01 | 25 | 0.53 ± 0.01 |
|  | J3 | 12 | 23.2 | 17 | 0.60 |
|  | 9-8 | 12 | 23.2 | 17 | 0.60 |
|  | 9-4-2 | 11 | 24.5 ± 0.2 | 73 | 0.58 ± 0.03 |
|  | **all** | **59** | **24.2 ± 0.2** | **32** | **0.57 ± 0.02** |

Bioluminescence rhythms of *per^01^* flies carrying the *plo* reporter gene and either wild-type PER or M560D PER encoding constructs were recorded as described in Materials and Methods. Flies were kept in 12 hr: 12 hr LD cycles at 25°C for the entire length of the experiment (5 days). Period values and rhythmicity was determined by performing an FFT-NLLS analysis with the raw data. Flies with rel-amp errors < 0.7 and period values of 24 ± 2 hr were considered as rhythmic (Materials and Methods).
